# Exploring the Extracellular Macromolecular Composition of Crude Extracts of *Penicillium rubens* Strain 212 for Elucidation Its Mode of Action as a Biocontrol Agent

**DOI:** 10.3390/jof6030131

**Published:** 2020-08-10

**Authors:** Maria Carreras, Eduardo A. Espeso, Alba Gutierrez-Docio, Silvia Moreno-Fernandez, Marin Prodanov, Maria Dolores Hernando, Paloma Melgarejo, Inmaculada Larena

**Affiliations:** 1 Departamento de Protección Vegetal, SGIT-INIA, Carretera de La Coruña 7, 28040 Madrid, Spain; carreras.maria@inia.es (M.C.); pmelgarejo@mapa.es (P.M.); 2 Departamento de Biología Celular y Molecular, Centro de Investigaciones Biológicas Margarita Salas-CSIC, Ramiro de Maeztu 9, 28040 Madrid, Spain; eespeso@cib.csic.es; 3 Departamento de Química Física Aplicada, Universidad Autónoma de Madrid (UAM), Ciudad Universitaria de Cantoblanco, 28049 Madrid, Spain; alba.gutierrez@uam.es (A.G.-D.); silvia.moreno@uam.es (S.M.-F.); marin.prodanov@uam.es (M.P.); 4 Departamento de Medio Ambiente y Agronomía, SGIT-INIA, Carretera de La Coruña 7, 28040 Madrid, Spain; hernando.dolores@inia.es

**Keywords:** biocontrol, biological control agents, extracellular proteins, metabolites, proteome

## Abstract

*Penicillium rubens* strain 212 (PO212) acts as an inducer of systemic resistance in tomato plants. The effect of crude extracellular extracts of PO212 on the soil-borne pathogen *Fusarium oxysporum* f. sp. *lycopersici* has been evaluated. Evidence of the involvement of soluble, thermo-labile, and proteinase-inactivated macromolecules present in PO212 crude extracts in the control of *Fusarium* vascular disease in tomato plants was found. Proteomic techniques and the availability of the access to the PO212 genome database have allowed the identification of glycosyl hydrolases, oxidases, and peptidases in these extracellular extracts. Furthermore, a bioassay-guided fractionation of PO212 crude extracellular extracts using an integrated membrane/solid phase extraction process was set up. This method enabled the separation of a PO212 crude extracellular extract of seven days of growth into four fractions of different molecular sizes and polarities: high molecular mass protein fraction >5 kDa, middle molecular mass protein fraction 5–1 kDa, low molecular mass metabolite fraction, and nutrients from culture medium (mainly glucose and minerals). The high and middle molecular mass protein fractions retained disease control activity in a way similar to that of the control extracts. Proteomic techniques have allowed the identification of nine putatively secreted proteins in the high molecular mass protein fraction matching those identified in the total crude extracts. Therefore, these enzymes are considered to be potentially responsible of the crude extracellular extract-induced resistance in tomato plants against *F. oxysporum* f. sp. *lycopersici*. Further studies are required to establish which of the identified proteins participate in the PO212’s action mode as a biocontrol agent.

## 1. Introduction

Soil-borne pathogenic fungi such as *Fusarium oxysporum* Schlechtend.:Fr. cause various plant diseases that are responsible for severe economic and agricultural losses in the world as they affect horticultural crops such as peppers, tomatoes, and cucumbers [1,2]. Chemical pesticides, such as pyrimethanil, carboxin, and benomyl, have been largely used to reduce the damage caused by these fungi [3]. However, the indiscriminate use of chemical pesticides affects the agriculture sustainability due not only to environment pollution, but also to public health problems and the possible development of resistance by phytopathogenic fungi [4]. 

Reduction or removal of chemical pesticide applications in agriculture is a global objective. A promising approach to management of soil-borne diseases is the use of new approaches such as biological control using microorganisms that antagonize phytopathogenic fungi either alone or integrated with reduced doses of chemicals, resulting in a minimization of the impact of chemicals on the environment [5,6,7]. In particular, biological control agents (BCAs) can play a key role in protecting crops against *Fusarium* spp. [8]. So far, different bacterial species from the genus *Pseudomonas, Serratia*, *Bacillus*, and *Streptomyces* have been effective in controlling wilt caused by *Fusarium* [9,10]. Regarding fungi, species of the genera *Trichoderma, Gliocladium, Penicillium*, and non-pathogenic strains of *Fusarium* are among the antagonistic fungi that have been effective against *Fusarium* [8,10].

BCAs may have different modes of action to control plant pathogens [11]. Among them, the main ones are competition, hyperparasitism, production of antibiotics and secondary metabolites, and induced resistance and priming [12]. Understanding the mechanisms involved in the interaction between BCA, the host plant, and the target fungal pathogen is essential to improve the use of these microorganisms in agriculture [13].

Currently, the most important efforts in biocontrol research focuses on plant–microbial interactions, which have led to an increase in the number of publications on the induction of BCA-mediated plant defense responses [5,14]. The two groups of BCAs that have received most attention in this field are *Trichoderma* spp. and *Pseudomonas* spp. In plants, the defense mechanisms are triggered by elicitors that are host plant- or microorganism-derived molecules. Among these elicitors, both microorganism-derived proteins and secondary metabolites have been identified. In this sense, it has been demonstrated that the inoculation of *Trichoderma* spp. onto roots induced the upregulation of several proteins and enzymatic activities involved in the defense of the plant, a situation that typically would happen when the plants are under attack by a pathogen [15]. There are also many secondary metabolites involved in hyperparasitism or *Trichoderma* spp. antibiosis that can trigger plant resistance [12]. On the other hand, culture filtrates of *Trichoderma virens* strains which are effective as BCAs have been shown to stimulate terpenoid production at significantly higher levels in cotton plants than those from non-effective strain culture filtrates or culture media alone [16].

One of these fungal BCAs is the soil-borne fungus *Penicillium rubens* strain 212 (PO212). This strain was formerly known as *Penicillium oxalicum* but was reclassified in 2016 [17]. The beneficial effect of PO212 on plant diseases suppression is well known and documented by many articles devoted to the biocontrol efficacy of PO212 against a wide range of important plant pathogens infecting different horticultural crops [18,19,20,21,22,23,24]. In previous studies, it was demonstrated that PO212 utilizes the induction of the plant defense response as mechanism of disease control caused by *F. oxysporum* f. sp. *lycopersici* (Sacc.) W.C. Snyder & H.N. Hansen (FOL) in tomato plants [25,26]. In these works, PO212 induced systemic morphological changes in tomato plants infected by FOL [26]. The possible role of pathogenesis-related (PR) proteins (chitinases, β-1,3-glucanases, and PR-1) has been studied, but no evidence of involvement of these proteins in the biocontrol process was found [27]. Pascual et al. [28] also showed that during PO212–plant–pathogen interaction, a filtered Morton’s medium after PO212 growth was effective to reduce disease caused by FOL in tomato plants. In this interaction, several different classes of extracellular compounds produced by PO212 could act in the biocontrol activity against the soil-borne pathogen FOL, but the chemical nature of these extracellular compounds is unknown. 

Due to the relevance of extracellular metabolites of PO212 extracts, that could include proteins and other low molecular mass metabolites in its biocontrol mechanisms, this work was focused on (i) the evaluation of the biocontrol activity of conidia-free PO212 crude extracts (CE) and some fractions obtained from them, and (ii) the chemical characterization of CE and fractions obtained from them that could be involved in the biocontrol activity of PO212 against FOL. Consequently, preparative separation techniques and proteomic and metabolomic techniques were used for their characterization.

## 2. Materials and Methods 

### 2.1. Microbial Cultures and Growth Conditions

The isolate 212 of *Penicillium rubens* (formarly *P. oxalicum*) (hereafter PO212) (ATC 201888) was stored in both 20% (*v*/*v*) glycerol (long-term storage) at −80 °C and on potato dextrose agar (PDA; Difco, Detroit, MI, USA) at 4 °C in the dark (short-term storage). Dried conidia of *P. rubens* isolate 212 (hereafter PO212) were produced in a solid-state fermentation system and dried as previously described [29]. To prepare a conidial suspension of PO212 (1 × 10^7^ conidia g^−1^ substrate), PO212 conidia were rehydrated in sterile distilled water (SDW) using a rotatory shaker at 150 rpm for 2 h. The day before treatment, viability of PO212 conidia was estimated by measuring their germination as previously described [29]. For each replicate (three by sample type), the germination of 50 randomly selected conidia was counted and was calculated and expressed as percentage [30]. The viability of these conidia was always greater than 90%. One third of PO212 conidia were killed by autoclaving for 30 min at 1 g cm^−2^ and 120 °C and called aPO212 (autoclaved conidia). 

Isolate 1A of *F. oxysporum* f. sp. *lycopersici* (Sacc.) W. C. Snyder & H. N. Hans. (FOL1A) was used as a pathogenic strain. It was provided by Dr. Cristina Moyano from the Laboratory for Assessment of Variety, Seed and Nursery Plants, INIA (Madrid, Spain). The isolate was stored at 4 °C in tubes containing sterile sand. For mycelial production, FOL1A from sterile sand was grown on Czapek Dox agar (CA) (Difco Laboratories, Detroit, MI, USA) in darkness at 25 °C for seven days. Microconidial inoculum of FOL1A was produced as described elsewhere [31]. The conidial concentration was determined using a hemocytometer and adjusted to 10^6^ microconidia mL^−1^.

### 2.2. Production of PO212 Conidia and Crude Extracts from Submerged Cultures

PO212 conidia and crude extracts were also produced in liquid-state fermentation using Morton liquid medium, as described by [32]. Morton A and B media were prepared as previously described [31]. Erlenmeyer flasks of 250 mL containing 50 mL of Morton A medium were inoculated with one mL of conidia suspension of PO212 (1 × 10^7^ conidia mL^−1^) taken from the seven-day-old culture on PDA plates and incubated at 25 °C on an orbital shaker (150 rpm) for 24 h in darkness. After 24 h of incubation, the conidia were filtered through sterile Whatman No. 1 filter paper, washed twice with SDW, and transferred to other 250 mL sterile flasks containing Morton B medium. Conidia were then incubated as above for 6 and 13 additional days in darkness. From each culture, mycelium was collected by filtration through 22–25 µm filter Miracloth (Merk Milipore, Billerica, MA, USA). Filtered supernatant was centrifuged (14,040× *g* at 4 °C for 20 min) (SORVALL^®^ RC5C Plus, Kendro Laboratory Products, Newtown, CT, USA) and pellet and supernatant were collected separately. The pellet was resuspended in SDW and was named “submerged conidia culture” of 7 and 14 days (SCC7 and SCC14, respectively). The supernatant was again filtered using a vacuum filter unit (Sartorius™ Sartolab™ RF/BT 0.22 µm pore size polyethersulfone membrane, Goettingen, Germany) to remove any conidia. The conidia-free filtrate from PO212 submerged culture of 7 and 14 days of growth was called “crude extract” of 7 and 14 days (CE7 and CE14, respectively). CEs were either used directly or autoclaved and brought to the desired concentration by SDW. Autoclaving was carried out for 30 min at 1 g cm^−2^ and 120 °C to inactive thermo-labile compounds present in CE7 and CE14. These extracts were called aCE7 and aCE14 (autoclaved).

### 2.3. Evaluation of Control Activity of PO212 Conidia and Crude Extracts from Submerged Cultures Against FOL1A in Tomato Plants

To characterize those compounds produced by PO212 and involved in its control activity against FOL1A, diverse growth chamber experiments were carried out on tomato plants (see also [33]) cv. “San Pedro”. Seeds were sown in sterile trays (1200 mL) containing an autoclaved mixture of vermiculite (Termita; asfaltex. S.A. Barcelona. Spain) and peat (Gebr. BRILL substrate; GmbH & Co. KG) (1:1, *v*/*v*) (autoclaved for 1 h at pressure of 1 kg cm^−2^ and temperature of 120 °C, during three consecutive days) and maintained in a growth chamber at 22–28 °C, under fluorescent light (100 µE m^−2^ s^−1^ and 16 h photoperiod) and 80–100% relative humidity for 3–4 weeks [31]. 

Four types of assays were carried out. For all assays, the tomato seedlings (2–4 true leaves) were watered with the different treatments seven days before they were transplanted into flasks. All treatments including conidia were done in the proportion 1 × 10^7^ conidia g^−1^ of seedbed substrate (vermiculite: peat, 1:1, *v*/*v*). Those treatments not including conidia (CEs, Morton’s medium, digested buffer) were done by watering with 60 mL of each treatment kg^−1^ of seedbed substrate. Table 1 describes the applied treatments in each assay. Firstly, the activity of PO212 SCC and CE against FOL1A in tomato plants was evaluated and compared with that of PO212 from solid-state fermentation (A1). CEs were autoclaved to assess the involvement of thermo-labile compounds in the disease control activity of PO212 CEs. Next, the optimal concentration of CE that produced the highest activity of control against FOL1A in tomato plants was determined (A2). Therefore, three different dilutions of CE in SDW were used. In order to determine a possible involvement of proteins compounds present in CE in the activity against FOL1A, a third type of assay was made (A3). To this end, a CE sample was mixed (1:4, *v*/*v*) with 5× digestion buffer (250 mM TRIS-HCl pH 7.5 and 25 mM CaCl_2_) and 5× proteinase K (final concentration 0.1 mg mL^−1^) (E.C.3.4.21.64, Sigma-Aldrich) following an incubation at 50 °C for 1 h. This digested sample was called d-CE. Finally, in the A4 assay, the optimal conditions of storage of CE for subsequent uses were studied. For this end, CE was kept at −20 °C and −80 °C for 24 h, and after that, it was allowed to thaw under agitation in a rotary shaker (150 rpm) at room temperature (RT) before its application. 

For seedling assays (Figure S1), one week after treatments, tomato seedlings with two to four true leaves were transplanted from seedbeds into 100-mL flasks containing 125 mL sterile Hoagland Nº 2 solution [31,34]. Five replicate flasks, each containing four plants, were used per treatment. The flasks were placed in a randomized complete block design in a growth chamber at 22–28 °C, under fluorescent light (100 µE m^−2^ s^−1^ and 16 h photoperiod) and 80–100% relative humidity for four to five weeks. Inoculation with the pathogen was performed by adding a microconidial suspension of FOL1A in SDW to each flask just before transplanting, to a final concentration of 1 × 10^6^ microconidia mL^−1^.

Prior to transplanting, populations of PO212 in plants and seedbeds treated with PO212 from solid- and liquid-state fermentation (PO212 and SCC, respectively) were estimated as number of colony-forming units (cfus) g^−1^ of fresh substrate or root, respectively, as previously described [22]. Populations of PO212 were also estimated in plants and seedbeds treated with SDW.

Every seven days, during four or five weeks after transplanting, the following parameters were recorded: nutritive solution consumption per plant, flask and day; leaves number per plant; and disease severity according the scale previously described [18]. At the end of each assay, the area under the disease progress curve (AUDPC) was calculated as described in [35]. Fresh weight of roots and aerial parts per flask were recorded as well. To confirm that plants with symptoms were infected with FOL1A, they were transferred to humid chambers, and the presence or absence of mycelium of the pathogen was determined in the basal stems of the plants after incubation at 25 °C for five days. 

### 2.4. Test of Antifungal Activity of PO212 Crude Extracts (CE7) Against FOL1A

The effect of PO212 CE7 on the FOL1A mycelial growth and sporulation density was studied in in vitro assays on CA-containing Petri dishes that were supplemented with the following concentrations of CE7: 2.5, 12.5, 25, 50 and 80%. Then, these Petri dishes were inoculated at the center with mycelial plugs (0.6-cm in diameter), cut from the colonies of seven-day-old FOL1A actively growing on CA. As a control, CA plates without CE7 (0%) were taken. The inoculated plates were then incubated at 22 °C for seven days in darkness. The FOL1A growth rate (mm day^−1^) was calculated by taking two perpendicular colony diameter measurements each day until seven days of growth. At the end of the assay, the sporulation density was calculated as previously described [36]. Five CA plates were used for each CE7 concentration. The assay was repeated at least twice.

The effect of CE7 on conidial germination and germ tube growth of FOL1A was evaluated as previously described (Section 2.1). Briefly, 20 µL of FOL1A suspension conidia (10^6^ conidia mL^−1^) in CB were added on 20 µL of different concentrations of CE7 (2.5, 12.5, 25, and 50%) in CB. As a control, 20 µL of SDW were applied instead of the CE7. All concentrations included four repetitions (drops). For each drop, the germination of 50 randomly selected conidia was counted and the length of the 25 germ tubes was measured.

### 2.5. Analysis of CE7 Low Molecular Mass Secondary Extracellular Metabolites

CE7 was submitted to a chemical characterization with the objective to identify the main bioactive low molecular mass secondary extracellular metabolites, which could be responsible for the biocontrol activity of PO212.

For analysis of low molecular mass secondary extracellular metabolites, CE7 was submitted to the clean-up procedure based on a dispersive solid phase extraction (SPE) assisted with ultrasounds [37]. In brief, each sample (1 g) was extracted with 5 mL of acetonitrile (ACN) (CH_3_CN, Scharlab, Barcelona, Spain). A mixture of 3.25 g of anhydrous magnesium sulphate (MgSO_4_), sodium chloride (NaCl), sodium hydrogen citrate sesquihydrate (Na_4_C_12_H_18_O_17_), and sodium citrate tribasic dehydrate (Na_3_C_6_H_9_O_9_) (in an equivalent proportion 8:2:2:1 of weight) was added to facilitate the clean-up of the sample. After shaking and centrifugation for 5 min at 3170 g and 4 °C, a low temperature precipitation procedure was carried out for 1 h at −20 °C. An aliquot of the extract (3 mL) was transferred into a 15 mL polypropylene centrifuge tube with 100 mg of a mixture containing anhydrous MgSO_4_, and the sorbents (PSA) and octadecyl silane (C18) (in an equivalent proportion 6:1:1 of weight) were added and shaken again for 1 min, followed by centrifugation for 5 min at 3170 g. An aliquot of 250 µL of supernatant was diluted with 750 µL Milli-Q grade water (Merck Millipore, MA, USA) and transferred into a vial. The extracts were filtered through a Nylon membrane filter with a pore size of 0.22 µm (Phenomenex, Torrance, CA, USA) before analysis.

A high-performance liquid chromatograph coupled to a hybrid quadrupole time-of-flight-tandem mass spectrometer (HPLC–ESI–QTOF–MS/MS) was used for the non-target analysis of metabolites. The HPLC system (Agilent 1290 Series) was equipped with a C8 reversed-phase analytical column (100 mm × 3 mm, i.d. and 3 µm particle size, 100 Ǻ porosity) (Luna Omega Polar C18, Phenomenex, Torrance, CA, USA). The mobile phase was composed by A (Milli-Q-grade water and 0.1% formic acid) and B (ACN and 0.1% formic acid). The elution gradient was as follows: 20% B (2 min), 13 min linear gradient to 100% B (2 min), and 2.5 min post-run time back to the initial conditions. The flow rate was 0.3 mL min^−1^ and the injection volume was 10 µL. The QTOF–MS (Agilent 6530 Series Accurate Mass QTOF–MS, Agilent Technologies, Santa Clara, CA, USA) was operated in the 2 GHz High-Resolution (HR) mode and in positive ionization mode. Ions were generated by electrospray using a Dual Spray Agilent Jet Stream ion (ESI) source. The ESI parameters were as follows: interface temperature, 300 °C; heating gas flow, 10 L.min^−1^; drying gas flow, 10 L.min^−1^; capillary tension, 4 kV; and nebulizer pressure, 12 psi. The system was calibrated over a mass range of 0−1700 *m/z*. An internal lock mass mixture, containing purine (*m/z* 121.0509) and HP-0921 (*m/z* 922.0098) was constantly infused for mass correction. 

Analysis of samples was performed in full scan mode; screening of metabolites comprised a retrospective study using MassHunter™ Qualitative Analysis (B.07) software (https://www.agilent.com/en/products/software-informatics/mass-spectrometry-software). Tentative identification of metabolites with a molecular mass range less than 1000 Da was based on the measurement of the exact mass of at least two ions, preferably, a pseudo-molecular ion [M + H]^+^ and a fragment ion with structural characteristics in a mass error < ±5 ppm.

### 2.6. Bioassay-Guided Fractionation of PO212 Crude Extract (CE7)

As a second step of the chemical characterization of the possible bio-active compounds from CE7, a preparative bioassay-guided fractionation of CE7 was carried out using an integrated pressure-driven ultrafiltration (UF)/solid phase extraction (SPE) procedure.

**Figure 1:**
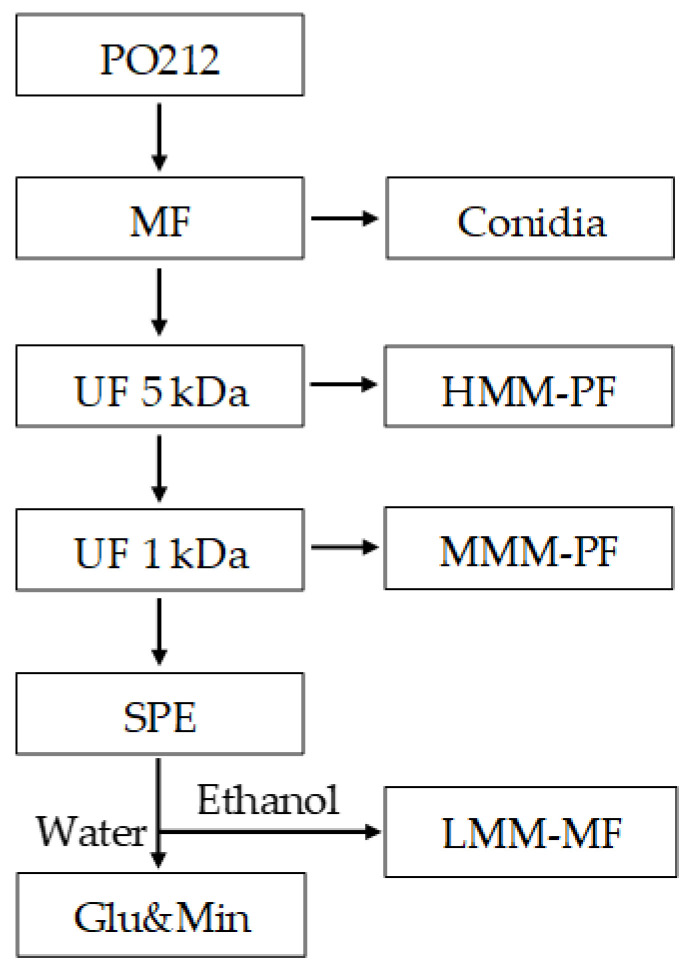
General diagram of the fractionation pathway of crude extract (CE7) of *Penicillium rubens* strain 212 (PO212) by tangential-flow ultrafiltration (UF) and solid phase extraction (SPE). MF: microfiltration; HMM-PF, high molecular mass (MM) protein fraction, MMM-PF, middle MM protein fraction, LMM-MF, low MM metabolic fraction, Glu&Min: glucose and mineral enriched fraction.

As shown in Figure 1, the general fractionation pathway consisted of the following main steps: separation of fungal conidia by a vacuum-driven dead-end microfiltration (MF) on a 0.22 µm pore size membrane filter, separation of high molecular mass (MM) protein fraction (HMM-PF) by a pressure-driven tangential flow UF on a 5 kDa MM cut-off (MMCO) membrane, separation of middle MM protein fraction (MMM-PF) by a pressure-driven tangential-flow UF on a 1 kDa MMCO membrane and separation of low MM metabolic fraction (LMM-MF) and nutrients from culture medium culture (mainly glucose and minerals) (Glu&Min) by a SPE on an adsorbent polymer resin.

#### 2.6.1. Preparative Pressure-Driven Tangential-Flow Membrane UF

A HMM-PF was separated first from the clarified CE7 extract by a 5 kDa MMCO regenerated cellulose membrane (Millipore, Merck, Darmstadt, Germany) (Figure 1). The membrane had a spiral wound shape, 0.54 m^2^ total filtration surface, and was inserted into a model Prep-scale 6 housing. The housing was connected to a low-pressure UF unit as previously described by Silván et al. [38]. UF was carried out in a continuous concentration mode at a transmembrane pressure (P_TM_) of 0.8 bar. An amount of 8.36 L of the clarified PO212 extract was concentrated to 0.7 L. Recovery of macromolecules was carried out by diafiltration of the concentrate with 4.9 L of demineralized water. The purified HMM-PF was stored at −20 °C until its use. In the other hand, a total amount of 7.7 L of ultrafiltrate was obtained and used for the next UF step. 

Next, a middle MM protein fraction (MMM-PF) (Figure 1) was separated from the permeate from the 5 kDa MMCO membrane using a 1 kDa MMCO membrane (Pall Co., New York, USA) with the following technical characteristics: flat sheet modified polyethersulfone (Omega™), cassette-type membrane with a total filtration surface of 0.37 m^2^. The cassettes were inserted in a stainless steel housing, model Centramate from the same company, and connected to the same low pressure UF unit, as already previously described by Silván et al. [38]. UF was carried out in a continuous concentration mode at a PTM of 0.6 bar. An amount of 7.7 L of the permeate from the 5 kDa MMCO membrane was concentrated to 0.7 L. Recovery of MMM-PFs was carried out by diafiltration of the 0.7 L of the concentrate with 7.7 L of demineralized water. The purified MMM-PF was stored at −20°C until its utilization. Moreover, 7 L of a new highly ultrafiltrated fraction were obtained and used for SPE fractionation. 

#### 2.6.2. Preparative SPE

The highly ultrafiltrated permeate obtained from the 1 kDa MMCO membrane was further submitted to a SPE. 180 mL of PAD610 resin (macroporous polyacrylic crosslinked with divinylbenzene polymer) from Purolite Co. (King of Prussia, PA, USA) were packed in a 0.5 L glass tube provided with a fritted glass disk at the bottom to retain the resin and a Teflon valve. Four aliquots of 2 L each were loaded to the resin at a flow rate of 45 mL min^−1^ and the permeates obtained from them were recovered as a glucose and mineral-enriched (Glu&Min) fraction (Figure 1) and stored frozen until their use. After loading of each aliquot, the resin was diafiltrated with demineralized water until 0% of total soluble substances (TSS) were measured (by refractometry) at the exit of the column. This diafiltration water was added to the Glu&Min fraction. Adsorbed compounds were eluted with 0.5 L of 96% ethanol. The ethanol was recovered by vacuum distillation at 30 °C and the resulting aqueous fraction, enriched in low MM metabolite fraction (LMM-MF), was stored frozen until its utilization.

### 2.7. Evaluation of Disease Control Activiy of Purified Fractions from CE7 

After the fractionation of CE7 described in Section 2.6, two types of assays in growth chambers were carried out to evaluate the activity on disease caused by FOL1A in tomato plants of all purified fractions. Each experiment was repeated at least twice such as previously described (Section 2.3).

The following control treatments were carried out in all assays: (i) plants inoculated with FOL1A and treated with a PO212 conidial suspension (1 × 10^7^ conidia g^−1^ substrate); (ii) plants inoculated with FOL1A and treated with Morton B medium (60 mL kg^−1^ of substrate); (iii) plants inoculated with FOL1A and treated with SDW (60 mL kg^−1^ of substrate); and (iv) plants not inoculated with FOL1A and treated with SDW (60 mL kg^−1^ of substrate). 

Initially, the activity of all purified fractions was evaluated on disease control caused by FOL1A in tomato plants. The impact of the application of each of these fractions on the growth of tomato plants not inoculated with the pathogen was also evaluated. Those fractions with the highest activity against FOL were selected for the second type of assay. Aliquots of selected fractions, including CE7, were freeze-dried in a Cryodos-80 freeze-drier (Telstar S.A., Madrid, Spain) operating at 1.5 mbar and −80 °C for 24 h. After freeze-drying, samples were stored at RT until their use. 

### 2.8. Multidimensional Liquid Chromatography Mass Spectrometry (nLC-MS) Identification of Proteins in Total and Fractionated CEs

Analysis of extracellular proteins from active fractions was carried out as follows. Proteins were precipitated following the protocol as described elsewhere [39] using trichloroacetic acid (TCA) (9:1, *v*/*v*). The total protein extract was conserved at −20 °C until further characterization. Protein samples prepared in rupture buffer were fractionated with sodium dodecyl sulfate –polyacrylamide gel electrophoresis (SDS–PAGE) following the protocol as described elsewhere [40]. This material was analyzed by the Proteomics facility at the CIB (CSIC, Madrid, Spain). Samples were hydrolyzed by trypsin and the peptides obtained after hydrolysis were separated and analyzed for their molecular masses using a nLC-MS/MS (LTQ Orbitrap Velos, Thermo Scientific). Candidate proteins were identified using available *P. rubens* protein databases at NCBI (National Center for Biotechnology Information). Sample preparation for nLC-MS was as previously described [41]. The mass spectrometry data from three or two biological replicates were analyzed searching against *P. rubens* Wisconsin strain 54-1255 [42] database (12,771 protein sequences), using the Sequest search engine through Proteome Discoverer (version 1.4.1.14, Thermo Scientific) using default parameters. This fungus was formerly known as *Penicillium chrysogenum* but was reclassified in 2011 [43]. In the search parameters, cysteine carbamidomethylation and methionine oxidation were established as fixed modifications. The tolerance of the selection of precursors and product ions was set at 10 ppm and 0.5 Da, respectively. The identification of peptides was validated by the Percolator algorithm using a *q* value ≤ 0.01 [44]. 

The results were analyzed searching against PO212 genome (INIA’s database) using the NCBI’s amino acid and nucleotide sequence search tools (Blastp and tBlastn). The PO212 genome has been sequenced by INIA group and it will be published elsewhere. The subsequent identification of the proteins in active fractions was performed by the search engine https://www.uniprot.org/ and the physicochemical characterization (number of amino acids, molecular mass, and isoelectric point) of the identified proteins was theoretically determined (https://www.expasy.org). All identified proteins from each sample were classified into main metabolic pathways using the OG classification against the EggNog database [45], and classification into Gene Ontology analysis of candidates was carried out using Blast2go v5.0. The subcellular location of identified proteins was predicted based on SignalP 5.0 [46].

### 2.9. Data Analysis

Data were analyzed by analysis of variance (ANOVA) using Statgraphics R Centurion XVI, version 16.1.03. When variances were not homogeneous, the data from AUDPC and disease severity (%) were transformed to log_10_x+1 to improve their homogeneity before analysis. Germination percentage and sporulation data from in vitro tests were converted to arcsine transformation. The Student–Newman–Keuls multiple range test was used for comparison of means [47] when *F*-test was significant at *p* ≤ 0.05.

The AUDPC was calculated using the disease severity percentage data [35]:

*AUDPC* = Σ_*i*=1_^*n*−1^[(*t*_*i*+1_ − *t_i_*)(*y_i_* + y_i+1_)/2] (1)

where *t* is the number of days from transplanting to the end of the assay, *y* is the disease severity (%) for each plant, and *n* is the number of samples. 

All the experiments were repeated twice. When the replication confirmed the first result, only results from the first set of experiments are reported and discussed here.

## 3. Results

### 3.1. Control Activity of PO212 Conidia and Crude Extracts from Submerged Cultures Against FOL1A in Tomato Plants

Various types of assays were conducted in growth chambers in order to verify the involvement of extracellular and thermo-labile compounds secreted by PO212 in the culture media in their activity against FOL in tomato plants. Several controls were established prior to the evaluation of crude extracts (CE) from cultures of PO212 in biocontrol. The control plants, i.e., plants inoculated with FOL1A and treated with SDW (C1) were characterized by higher disease severity and incidence which increased significantly with increasing time of infection compared to healthy treated seedlings (plants treated with SDW and not inoculated with the pathogen, C2). Tomato plants showed as main disease symptoms wilting of stems and leaves in all assays. PO212 was not detected in the seedbed substrate or in the rhizosphere of the plants treated with SDW (C) just before transplanting in any assay. PO212 was isolated from PO212-treated seedbed substrate before transplanting, at amounts ranging in the interval of 0.4–5 × 10^6^ cfu g^−1^ fresh substrate. The populations of PO212 in the rhizosphere of PO212-treated tomato plants prior to transplanting ranged from 4–6 × 10^6^ cfu g^−1^ of fresh root. However, a lower density of PO212 in the seedbed substrate (2) and 2.4 × 10^4^ cfu g^−1^ fresh substrate, respectively) and in the rhizosphere of tomato plants (5 and 8.9 × 10^3^ cfu g^−1^ fresh root, respectively) treated with SCC7 and SCC14 was quantified. Viability of PO212 conidia was higher than 95% in all assays prior its application in seedbeds, but viability of SCC7 and SCC14 was less than 70%.

In this context, filtered media from 7 and 14 days PO212 cultures on Morton’s medium, named as CE7 and CE14, reduced disease severity and AUDPC induced by FOL1A significantly (*p ≤* 0.05) (Table 2) when they were compared to those of the C1 control plants. The reduction in the disease severity varied between 48 and 80%, and that of AUPDC between 30 and 45%. These reductions were significantly (*p* ≤ 0.05) similar to those of the PO212 (Table 2). Autoclaved CE7 (aCE7) and CE14 (aCE14) were tested in these assays. A significant (*p* ≤ 0.05) reduction in disease severity was also observed in plants treated with aCE7 and aCE14 when compared to C1 control plants. However, this reduction was significantly (*p* ≤ 0.05) lower than that of the CE7 and CE14 treatments (Table 2). On the other hand, there was no significant reduction (*p* ≤ 0.05) in disease severity and AUDPC in tomato plants treated with SCC7, SCC14, and aPO212 compared to C1 control plants (Table 2). These results have shown that heat treatment by autoclaving is able to strongly reduce the disease control of PO212 conidia and its extracts against FOL1A. 

Moreover, none of the applied treatments had negative effects, such as reduction in nutritive solution consumption, fresh stem weight, and leaves number on the growth of tomato plants compared to the control plants C2 (Table 3), except for root weight, so all treatments significantly (*p* ≤ 0.05) reduced root weight compared to control plants C2 (Table 3). In conclusion, freshly prepared CE7 was selected to perform the following assays.

The optimal concentration of CE7 application was also evaluated (Table 4). Activity of PO212 crude extracts after growth for seven days in Morton’s medium (CE7) and their dilutions (1:1, 1:10, and 1:50) against disease caused by FOL1A in tomato plants at 36 days after transplanting and inoculation in two experiments carried out in growth chamber conditions. The results showed that only the direct use of CE7 significantly (*p* ≤ 0.05) reduced disease severity (32.2%) and AUDPC (approximately 22%) caused by FOL1A in tomato plants to the same extent as PO212. Any treatment, except PO212, significantly (*p* ≤ 0.05) reduced disease incidence (approximately 39% reduction) compared to C1 control plants. Then, the optimal conditions of application of the CE7 were 60 mL of undiluted CE7 by watering in the seedbed seven days before transplanting and inoculation with the pathogen.

**Figure 2:**
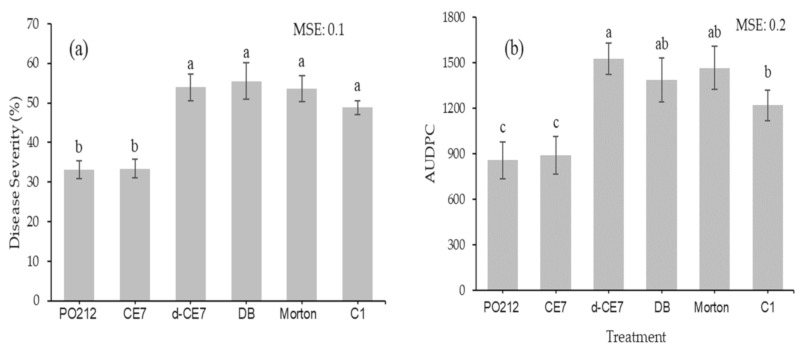
Effect of *Penicillium rubens* strain 212 (PO212) crude extract (CE7) after digestion with proteinase K (d-CE7) on the disease severity (**a**) and area under the disease progress curve (AUDPC) (**b**) caused by strain 1A of *Fusarium oxysporum* f.sp. *lycopersici* (FOL1A) in tomato plants at 29 days after transplanting and inoculation in growth chamber assay. CE7, PO212 CE after growing into Morton’s medium for 7 days; DB, Digestion buffer 5×; Morton, plants treated with Morton´s B medium; C1, control plants treated with sterilized distilled water and inoculated with FOL1A. See Table 1 for description of treatments. Data are mean value of two assays with ten replications (flasks) per treatment and four plants per replication. Data were subject to log_10_(x + 1) transformation to improve the homogeneity of variances before analysis. Means with the same letter in each parameter are not significantly different from each other (*p* ≥ 0.05) according to the Student–Newman–Keuls multiple range test. Vertical bars represent the standard error of the mean of ten replications. MSE is mean squared error of analysis of variance.

The involvement of proteins contained in the CE7 in the control activity by PO212 against FOL1A was studied. Treatment of CE7 with proteinase K (d-CE7) eliminated the capacity of CE7 to reduce disease severity and AUDPC caused by FOL1A in tomato plants (Figure 2). Only treatments with PO212 and CE7 significantly reduced (*p* ≤ 0.05) both the disease severity and the AUDPC in tomato plants inoculated with FOL1A compared to C1 control plants.

**Figure 3:**
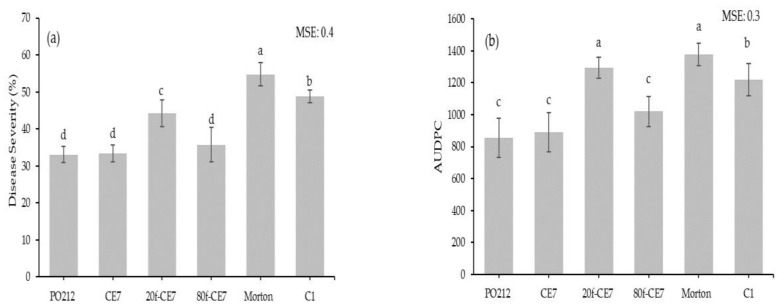
Activity of freezing of *Penicillium rubens* strain 212 (PO212) crude extract (CE7) at −20°C and −80 °C (20f-CE7 and 80f-CE7, respectively) on disease severity (**a**) and area under the disease progress curve (AUDPC) (**b**) caused by strain 1A of *Fusarium oxysporum* f.sp. *lycopersici* (FOL1A) in tomato plants at 36 days after its inoculation and incubation in growth chambers. Data are mean values of two assays with ten replications (flasks) per treatment and four plants per replication. ×; Morton, plants treated with Morton´s B medium; C1, control plants treated with sterilized distilled water and inoculated with FOL1A. See Table 1 for description of treatments. Data were subject to log_10_ (*x* + 1) transformation to improve the homogeneity of variances before analysis. Means followed by the same letter in each column are not significantly (*p* ≥ 0.05) different from each other according to the Student–Newman–Keuls multiple range test. Vertical bars represent the standard error of the mean of ten replications.

On the opposite to heat-treatment, freezing CE7 at −80 °C (80f-CE7) did not decrease the activity of this CE7 to reduce disease caused by FOL1A in tomato plants (Figure 3). A significant (*p* ≤ 0.05) reduction in disease severity and AUDPC was observed in plants treated with 80f-CE7 when compared to C1 control plants. These reductions were significantly (*p* ≤ 0.05) similar to those of the CE7 and PO212 treatments. However, the freezing at −20 °C (20f-CE7) decreased the activity of CE7 against FOL1A in tomato plants in comparison to treatments with PO212 and CE7 (Figure 3).

### 3.2. Test of the Antifungal Activity of PO212 Crude Extracts (CE7) Against FOL1A

A potential antifungal activity of CE7 on FOL1A was investigated by measuring the effect of different concentrations of the crude extract on mycelial growth, sporulation capacity, and conidial germination process of the pathogen in in vitro assays. The mycelial growth of FOL1A was not significantly (*p* ≤ 0.05) affected by the increase in the concentration of CE7 applied in the in vitro assays in CA plates (Table 5). However, sporulation of the pathogen was decreased for all concentrations tested after seven days of CE7 application. Increasing CE7 concentrations resulted in a decrease in FOL1A sporulation. A reduction of more than 90% of the sporulation ratio was observed for the highest concentration applied of CE7 (80%). Increasing CE7 concentrations resulted in an increase in the conidial germination rate and in the length of germ tubes of FOL1A. The highest concentration applied (80%) produced a 50% increase in the length of germ tubes and a 65% in conidial germination (Table 5). 

### 3.3. Analysis of CE7 Low Molecular Mass Secondary Extracellular Metabolites

So far it has been demonstrated that CE7 had the capability to retain disease control caused by FOL1A in tomato plants similar to that of PO212, and that the compounds potentially responsible for such control capability are partially inactivated by heat and digestion with proteases. 

As a first step in the chemical characterization of extracellular compounds present in the CE7, an extraction and characterization of the metabolites present in CE7 was done as described elsewhere (2.5).

**Figure 4:**
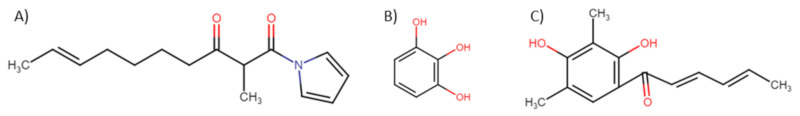
Chemical structure of the secondary metabolites identified in the PO212 crude extract (CE7). *N*-(2-methyl-3-oxodec-8-enoyl)-pyrrole (**A**), pyrogallol (**B**), and 1-[2,4-dihydroxy-3,5-dimethylphenyl]-hexadiene-2,4-one-1 (**C**).

Chemical characterization of CE7 metabolites was carried out by assigning molecular formula of those ions with signal intensities >103 and thresholds in mass error <±5 ppm. HPLC–MS allowed the tentative identification of three secondary metabolites classified as *N*-(2-methyl-3-oxodec-8-enoyl)-pyrrole, pyrogallol, and 1-[2,4-dihydroxy-3,5-dimethylphenyl]-hexadiene-2,4-one-1 (Figure 4).

The first of them had the highest signal at *m/z* 248.1641 that matched the molecular formula C_15_H_21_NO_8_ with a mass error of 1.8 ppm. Fragmentation of this ion generated two characteristic fragments via cleavage, either of the amide group or the C2′−C3′ bond, corresponding to the ions at *m/z* 181 and 125, respectively. The concordance of these structural characteristics with the elemental composition C_15_H_21_NO_8_ supports the assignation of a chemical structure corresponding to *N*-(2-methyl-3-oxodec-8-enoyl) pyrrole (Figure 4). The second peak showed higher signal at *m/z* 149.0213, which corresponds to the sodium adduct [M + Na]^+^ of pyrogallol. A fragment ion at *m/z* 99 was observed also, due to the loss of a carbonyl group, and a further loss of water producing an ion at *m/z* 165 from [M + Na]^+^. A characteristic loss of water due to the presence of two vicinal hydroxyl groups on the benzene ring was assigned to *m/z* 109. The mass accuracy of [M + Na]^+^ with a mass error of −2.49 ppm provided the basis for assessing structural assignment for this metabolite as pyrogallol.

Finally, a sorbicillin-related compound was assigned to the molecular formula C_14_H_16_O_3_ based on the determination of the accurate mass of the [M + H]^+^ at *m/z* 233.1179, with a mass error of −2.8 ppm, and the spectral features that provides structural information of the molecule. The compound was shown to be 1-[2,4-dihydroxy-3,5-dimethylphenyl]-hexadiene-2,4-one-1 (Figure 4). Structural elucidation relied on spectrum interpretation from ion detection at *m/z* 215, 205, and 165. A neutral loss of water followed by the loss of carbon oxide is consistent with the signals at *m/z* 215 and 205. Another feature that aids elucidation of this structure is the fragmentation, resulting in the loss of the alkyl side chain.

### 3.4. Bioassay-Guided Fractionation of PO212 Crude Extract (CE7)

As a second step of the chemical characterization of the possible bioactive compounds in CE7, a preparative fractionation of CE7 was carried out with the following objectives: (1) to separate proteins from low molecular mass secondary metabolites, (2) to separate proteins in two fractions with different molecular masses, (3) to separate low molecular mass metabolites from the Morton’s medium, and (4) to obtain enough amounts (in order of grams) of purified protein fractions and low molecular mass metabolites for their further chemical characterization and studies of their activity against FOL1A in tomato cultures at growth chamber conditions. A bioassay-guided fractionation of PO212 CE7 based on an integrated UF/SPE process was set up (see Section 2.6. Materials and Methods). This process enabled the separation of the CE7 into four fractions of different molecular sizes and polarities: HMM-PF >5 kDa, MMM-PF 5–1 kDa, LMM-MF, and Glu&Min.

### 3.5. Evaluation of Disease Control Activity of Purified Fractions from CE7 

Activity of different fractions obtained after bioassay-guided fractionation of PO212 crude extract (CE7) on the disease control caused by FOL1A and on the growth of tomato plants at 20 days after transplanting in growth chamber assays was evaluated. All untreated and inoculated with FOL1A tomato plants showed symptoms of *Fusarium* wilt (C1, Table 6 and Table 7) manifested as wilting of stem and leaves. No disease was observed in plants non-treated and non-inoculated with FOL1A (C2, Table 6 and Table 7). FOL was isolated from all diseased plants.

The viability of the PO212 conidia was higher than 95% at the time of application in seedbeds. PO212 was not detected in the seedbed substrate or in the rhizosphere of the plants treated with SDW just before transplanting. The PO212 population in the treated tomato rhizosphere just prior transplanting was approximately 5 × 10^6^ cfu g^−1^ of fresh root in both assays. PO212 was isolated from treated seedbed substrate before transplanting and was approximately 5.2–7.1 × 10^5^ cfu g^−1^ of fresh substrate.

Statistical analyses showed that all fractions derived from the bioassay-guided fractionation (HMM-PF, MMM-PF, Glu&Min, and LMM-MF) significantly reduced (*p* ≤ 0.05) disease severity and AUDPC caused by FOL1A in tomato plants, compared to those treated with SDW (C1) or Morton´s medium and inoculated with FOL1A (Table 6, rows with an asterisk), although only treatments with HMM-PF and MMM-PF reduced disease severity (74% in reduction) at a degree similar to those of PO212 and CE7 from which they derive. In terms of disease incidence, all fractions, except Glu&Min significantly reduced (*p* ≤ 0.05) the disease incidence compared to those of control (C1) or Morton´s medium and inoculated with pathogen (Table 6, rows with an asterisk). The reduction ranged from 30 to 70%.

On the other hand, application of HMM-PF and MMM-PF significantly increased (*p* ≤ 0.05) leaf number per plant and fresh stem and root weight compared to C1 (Table 6, rows with an asterisk), although an increase in nutritive solution consumption by the HMM-PF and MMM-PF was also observed (this increase was not significant (*p* ≤ 0.05) when compared to C1) (Table 6, rows with an asterisk).

In addition, the effect of application of the studied fractions on the growth of tomato plants non-inoculated with FOL1A was evaluated (Table 6, rows without an asterisk). The results showed that application of all studied fractions on the growth of tomato plants non-inoculated with FOL1A did not has a negative effect. These plants showed a significant reduction (*p* ≤ 0.05) in fresh stem and roots weight compared to C2. Only the treatment with PO212 significantly (*p* ≤ 0.05) increased all studied parameters (nutritive solution consumption, leaves number, fresh stem weight, and roots weight) compared to C2 (Table 6, rows without an asterisk).

Taking in consideration these results, both protein fractions, HMM-PF and MMM-PF, were selected as those fractions that retain disease control activity caused by FOL1A in tomato plants similar to that of CE7. For this reason, the effect of freeze-drying of HMM-PF and MMM-PF (called HMM-PF/L and MMM-PF/L, respectively) on the disease control activity of these fractions against FOL1A in tomato plants at 20 days after transplanting in growth chamber assays was evaluated (Table 7). The results showed that freeze-drying (freezing and dehydration) did not significantly reduce (*p* ≤ 0.05) the disease control activity (disease severity and incidence, and AUDPC) of both fractions in tomato plants inoculated with FOL1A when compared to the unfreeze-dried fractions (HMM-PF and MMM-PF) (Table 7). When growth parameters were evaluated, a significant (*p* ≤ 0.05) negative effect was observed in plants treated with CE7L and HMM-PF/L with respect to the same unfreeze-dried CE7 and HMM-PF, except for the nutritive solution consumption. The nutritive solution consumption in plants treated with CE7L and HMM-PF/L was similar when it was compared to the same unfreeze-dried CE7 and HMM-PF (Table 7).

### 3.6. nLC-MS/MS Identification of Proteins in Total and Fractionated CEs

**Figure 5:**
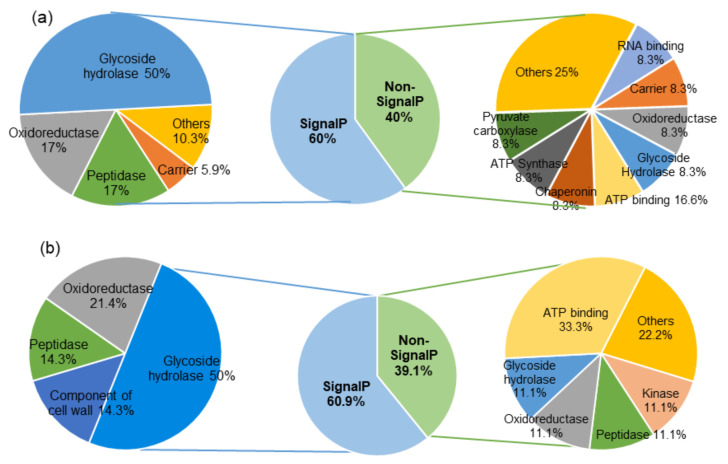
Proteome of the *Penicillium rubens* strain 212 crude extract after growth for seven days in Morton’s medium (CE7) (**a**) and the high molecular mass protein fraction (HMM-PF) (**b**) identified in predictive genome of PO212 by nLC-MS/MS. The identified proteins were distributed according to their corresponding function (according to UniProt).

Following the chemical characterization of CE7, the presence of extracellular proteins contained in CE7 were determined by nLC-MS/MS proteomics and identified using the *P. chrysogenum* Wisconsin protein database (Table S1). Possible orthologs were searched in the PO212 predicted proteome (Table S1). Similarly, predictions of protein functions of PO212 orthologs were comparable to *P. chrysogenum* Wisconsin proteins (Figure 5a, Table 8). By this approximate, a total of 30 fungal proteins were identified, of which 18 (60%) carried a signal peptide in their N terminal amino acid sequence, indicating that they might be actively secreted. Of the 18 putatively secreted proteins, the largest annotated group of proteins identified corresponded to glycoside hydrolases (GH) (50%) with a total of nine CAZymes (carbohydrate active enzymes) of GH families 3, 5, 15, 16, 17, 32, 43, and 72, followed by oxidoreductase (3) and putative protease (3) activity (Figure 5a, Table 8). 

On the other hand, 12 of the 30 proteins total (40%) detected in CE7 lacked a signal peptide (Figure 5a). Higher variability of functions was found among putatively non-secreted than secreted proteins (Figure 5a), as were proteins related to metabolic processes, oxidoreductases (g5740), and other different functions (Table 8).

Once HMM-PF and MMM-PF were identified as those that retain the disease control activity against FOL1A as the CE7 from which they derive, the pattern of proteins contained in HMM-PF and MMM-PF was analyzed by nLC-MS/MS proteomics. Proteins present in HMM-PF were identified using the *P. chrysogenum* Wisconsin protein database and the corresponding orthologs found in the predictive PO212 proteome (Table S2). A total of 23 proteins were identified. Searches determined that 60.9% (14) of detected proteins contained a signal peptide, indicating that they might be actively secreted (Figure 5b). Like in CE7, the largest annotated group of proteins identified were CAZymes (50%) with a total of seven proteins from different GH families 3, 15, 16, 17, 25, 43, and 47, followed by annotated enzymes with oxidoreductase (3) and proteolytic (2) activity (Figure 5b, Table 9). The remaining 39.1% (9) corresponded to proteins without signal peptide and were identified as GH family 32 (1), peptidases (1), oxidoreductases (1), ATP binding (3) and others (2) (Figure 5b, Table 9). 

In contrast, only two proteins were identified in MMM-PF, both without a peptide signal, which are g6856 and g7065 (Table S3). One of them (Pc20g11630/g6856) was also identified in both CE7 and HMM-PF. This protein is gamma-actin and probably represents a contamination in extracts due probably to its stability and relative quantity in cells [48]. The second detected protein, encoded by Pc21g10070/g7065, is a subunit of the ATP synthase. Due to the size of fractionation, both identifications may correspond to fragments of the corresponding full-length proteins. 

**Figure 6:**
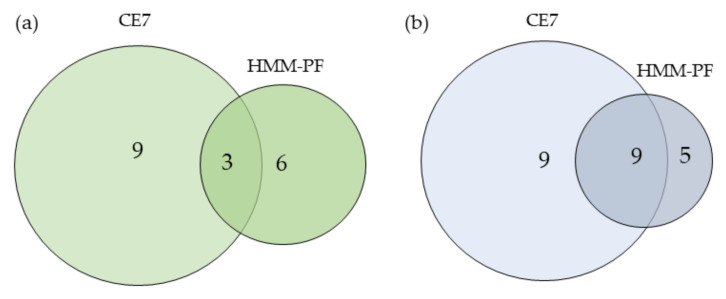
Comparative proteins lacked signal peptide (**a**) and with signal peptide (**b**) in the *Penicillium rubens* strain 212 crude extract after growth for seven days in Morton’s medium (CE7) and the high molecular mass protein fraction (HMM-PF) identified in predictive genome of PO212 by nLC-MS/MS. The identified proteins were distributed according to their corresponding function (according to UniProt). SignalP, Signal Peptide was predicted based on SignalP 5.0.

Figure 6 shows a comparative study of CE7 and HMM-PF exoproteomes. The number of common proteins with signal peptide to CE7 and HMM-PF was higher than for proteins without signal peptide. In the case of proteins without a signal peptide, only three common proteins among CE7 and HMM-PF were found (Figure 6a). They were g5740 (oxidoreductase activity), g7817 (ATP binding), and the gamma-actin g6856 (Table 8 and Table 9). In the case of proteins with signal peptide, nine common proteins were identified among CE7 and HMM-PF (Figure 6b). The largest annotated group of common proteins identified were the GH (with a total of five proteins from different GH families, all of them related to metabolic processes (g9789, g8238, g3615, g4058, and g9585). The other common proteins (2) were proteins assigned to the functional group of protein synthesis, degradation, and exchange (g9920 and g5776).

## 4. Discussion

The aim of this work was to evaluate the potential role of some extracellular macromolecules produced by *Penicillium rubens* strain 212 (PO212) that possibly are involved in the protective effect of this BCA against the soil-borne pathogen *F. oxysporum* f. sp. *lycopersici* (FOL), as well as to identify the chemical nature of these extracellular compounds implied in biocontrol efficacy against FOL. The results of the present study successfully demonstrated the involvement of a crude conidia-free culture (CE) obtained from PO212 in the efficacy of PO212 against FOL in tomato plants. The involvement of some compounds that are soluble, thermo-labile, and susceptible to digestion with proteases, namely proteins present in CEs, has been found to be responsible for the mode of action of PO212 against FOL. In parallel, an integrated UF procedure has been assayed to separate these compounds into two fractions with different molecular masses. Proteomic nLC-MS/MS techniques and the availability of the PO212 genome have allowed the identification of several glycoside hydrolases, oxidases, and peptidases in these CEs. Therefore, these enzymes are considered to be potentially responsible for the CE-activity against FOL in tomato plants in growth chamber assays. 

The management of persistent soil-borne pathogens through chemical means is inefficient as it involves high costs and cause environmental hazards. An ecofriendly approach in crop protection, capable of reducing the damage caused by fungal pathogens with several BCA, was reported to be effective in many crops [49,50,51]. In this sense, application of dried PO212 conidia (live conidia) has been already shown to trigger the plant defense response against FOL in tomato plants [25,26].

In this work, we found that conidia grown during 7 and 14 days in Morton’s liquid culture (CCS) was not so efficient against FOL1A in tomato plants as dried conidia of PO212 was. The main causes accounting for this difference are growth conditions that are similar to the natural habitat [52], more easily obtained aeration, and better access to nutrients [31] when conidia were grown in solid-state fermentation than in liquid cultures. In another study, Pascual et al. [32] also observed that conidia of PO212 from agar plates were more effective than submerged conidia against *Fusarium* wilt of tomato. Furthermore, the lower populations of PO212 observed in the rhizosphere of CCS-treated tomato plants (100 times) than those in the rhizosphere of dried PO212 conidia-treated tomato plants may be responsible for the observed lower disease control. In many biological control systems, lack of consistency has been associated with low populations of the antagonist [19,53]. Pascual et al. [32] showed that aerial and submerged spores of PO212 differ in hydrophobicity, viability, and efficacy against FOL. Differences in hydrophobicity have been shown to affect the viability of the BCA *Trichoderma harzianum*; aerially-produced spores were highly hydrophobic and showed longer viability after storage than submerged spores [54]. In this study, PO212 conidia from solid-state fermentation survived better than those from submerged culture (>90% survival for aerial spores and ≈ 70% for submerged conidia), similar to those described by Pascual et al. [32].

Among the available media, fungi are usually grown in liquid media in order to obtain extra and intracellular enzymes and secondary metabolites. All fungi need several specific nutrients or factors to grow and reproduce. The requirements for growth are generally less strict than for sporulation. Many studies show that the nature of the carbon and nitrogen sources, carbon concentrations, and the C/N ratios of the culture media strongly affect growth and sporulation of the fungi [55,56]. For example, PO212 displays a nitrate-deficiency (nit-) phenotype with poor colonial growth when nitrate is used as the main source of nitrogen [57]. Here the crude extract of PO212 (CE) after growing under nitrogen stress conditions (Morton’s medium) maintained the same disease control activity as the dried PO212 conidia. 

In this research, the activity of some soluble, thermo-labile, and protease-digestible compounds present in the crude extracts of PO212 (CE) against FOL in tomato plants, which can be stored at −80º C for long-term use, has also been demonstrated. CE7 (seven-day growth) was selected instead of CE14 (14-day growth) as it allowed a production of larger volume of CE to be produced in a shorter period. The findings of this study are also in agreement with many scientific reports which showed that application of a culture filtrate of other strains of *P. chrysogenum* triggers the defense response of the plant against other pathogens fungal in other crops [58,59]. In vitro culture assays showed that CE7 reduces FOL1A sporulation but increases mycelial growth and rate of germination of FOL1A, so these compounds responsible for the disease control could delay sporulation and, consequently, delay infection. Since the CE7 activity was reduced by heat treatment (autoclaving at 120 °C) and by digestion with proteases, it was deduced that the compounds involved in the disease control activity are most probably proteins or glycoproteins. The use of relatively novel tools to investigate the PO212 action mode, such as proteomic and metabolomics analyses, could help us to identify those compounds involved in this mode of action.

Considering the large number of different macromolecules and low MM metabolites produced by fungi, a bio-guided fractionation of its extracellular growth media is usually recommended before analysis by nLC-MS/MS. In this research, extracellular proteins of PO212 crude extract (CE7) were separated in two fractions containing proteins of high (HMM-PF) and middle (MMM-PF) molecular weights by an integrated membrane process. 50% of the extracellular proteins identified in CE7 were subsequently identified in the HMM-PF (>5 kDa). 

Proteomic studies are essential for characterization of the biocontrol process since the final genetic product responsible for the biocontrol properties (e.g., lytic enzymes and elicitors of plant resistance) can be directly identified. There are several reports on the use of proteomic tools in order to identify proteins associated with the control function in an effort to understand and determine the action mechanisms of BCAs [60,61]. In order to make better use of proteomic approximations, it is first advisable to know the genomic sequences of the living organisms under study and thus to have databases containing the masses of theoretical proteins that could be expressed by these organisms, so that they can be assigned an identity. However, there are options that help their treatment such as mass spectrometry [14,62]. The genome of PO212 has been sequenced and is being analyzed (Larena et al. unpublished). Preliminary analyses showed strong sequence conservation with that of *P. rubens* Wisconsin strain 54-1255, thus validating previous studies on the taxonomy of this BCA [17,63]. Here, it has served to support the proteomic searches using *P. rubens* Winsconsin protein database.

In this work, the proteins present in CE7 and MMM-PF were identified by nLC-MS/MS after tryptic digestion. In CE7, PO212 secreted proteins with signal peptide are predominantly CAZymes. Most of them are glycoside hydrolases (GHs, 50%). Oxidoreductases and peptidases were also identified (17%). It is noteworthy that most of these signal peptide proteins were also identified in HMM-PF. Many GH families are associated both with metabolism and mycoparasitism processes [64,65]. Since PO212 does not act through mycoparasitism, the production of these enzymes could be related to the primary metabolism of the fungus. Nonetheless, these GH are also known for having a central role in mycoparasitism acting as fungal cell wall degradative enzymes [66]. We can postulate that these PO212 GHs could confer an additional defensive advantage to a plant colonized by PO212. A similar conclusion was obtained by Lima et al. [65] with *Trichoderma harzianum* T1A. 

A serine endopeptidase (Pc21g14160/g5776) was also identified in both the CE7 extract and the HMM-PF. A possible ortholog has been related to the elicitation of plant defense responses by *Trichoderma virens* [16], being the most relevant extracellular protease related to the biocontrol process in *Trichoderma* sp. [16,67,68,69]. 

In addition to the serine endopeptidase, other PO212 proteins, such as GH and oxidoreductases, are detected in the absence of both the pathogen and the host plant. *T. harzianum* T1A is able to secrete proteins related to biological control as well as proteins that provide protection to the plant even in the absence of pathogen [65]. Our results could suggest that PO212 may constitutively express proteins possibly involved in biological control mechanisms as *T. harzianum*. 

Recent experimental studies revealed the secretion of a new type of secreted protein, lacking signal peptide, which support the existence of new secretion mechanisms regardless of the conventional endoplasmic reticulum secretory pathway [70]. Similarly, Nogueira-López et al. [71] analyzed the secretomes of *T. virens* and found evidence for non-conventional secretion mechanisms. A more recent analysis of the secretome of *Aspergillus fumigatus* and about ten other related species found that 64 fumigatus proteins (0.65% of the proteome) are secreted by non-conventional secretion mechanisms, compared to 598 (6.1% of the proteome) that are secreted through the of the conventional endoplasmic reticulum secretory pathway [72]. The biological function of these proteins needs to be further investigated. In our case, approximately 40% of CE7 and HMM-PF proteins are secreted by non-conventional secretion mechanisms, compared to 60% of protein secreted through the conventional endoplasmic reticulum secretory pathway. Furthermore, extracellular proteins were not detected in the MMM-PF, despite the fact that it showed disease control activity against FOL in tomato plants. The possible reasons may be either the existence of some type of not identified until now proteins without signal peptide, but that could have high disease control activity or that this fraction may contain polysaccharides that may be sensitive to heat. Extracellular proteins seem to participate in the antagonistic action of microorganisms against pathogenic fungi such as *Wickerhamomyces* *anomalus* (strain K) against *Botrytis cinerea* and *Penicillium expansum* on apples, *B. cinerea* on grapes, *Penicillium digitatum* on oranges, and *Colletotrichum gloeosporioides* on papaya [73,74,75]. In *Trichoderma virens*, an 18 kDa protein was isolated from its culture filtrate and identified as elicitor of the defense responses in cotton seedling roots [16]. Apart from extracellular proteins, some authors report that the secondary metabolites produced by other fungi such as *Trichoderma* have antifungal as well as mycoparasitism actions, defense response induction, and resistance in host plants [76].

On the other hand, metabolomics has enabled researchers to identify and quantify the compounds secreted by microorganisms. *Penicillium* species are important because of their widespread occurrence and ability to produce a diverse range of bioactive secondary metabolites with well-proven biological activities. For this, in the present study, analysis of the crude extracts of PO212 (CE7) by HPLC–ESI–QTOF-MS/MS screening was analyzed. This type of work allowed the identification of three low to medium polarity main metabolites secreted differently in crude extract (CE7) by PO212: *N*-(2-methyl-3-oxodec-8-enoyl) pyrrole, pyrogallol (1,2,3-trihydroxybenzene), and sorbicillin-related compound. These compounds are secondary metabolites of low to medium polarity and usually isolated in minimal quantities. Secondary metabolites were separated in a fraction of low molecular weights (LMM-MF) from CE7. However, although three secondary metabolites of low to medium polarity were identified in CE7 in minimal quantities, probably we were unable to identify secondary metabolites in the LMM-MF due to the same procedure used. This later process was developed for substances with high polarity and concentration in the sample.

Cantín et al. [77,78] isolated and identified *N*-(2-methyl-3-oxodecanoyl)-2-pyrroline from the culture medium of *Penicillium brevicompactum*, which the authors relate to in vivo anti-juvenile hormone activity [79]. The antioomycete activity of several *Metarhizium* extracts was also associated with previously isolated aurovertins, fungerin, *N*-(methyl-3-oxodec-6-enoyl)-2-pyrroline, and *N*-(methyl-3-oxodecanoyl)-2-pyrroline [80]. There are a number of both naturally occurring and synthetic pyrroles which possess plant protection properties. In particular, most of these compounds are effective against fungi and insects [78]. Pyrogallol-type phenolic compounds have three adjacent hydroxyl groups on the same benzene ring, which enables their easy oxidation or autoxidation and also allows metal ion chelation, both characteristics contributing significantly to a wide range of biological activities [81]. Finally, sorbicillinoids are secondary metabolites with anti-inflammatory and antimicrobial activities produced by filamentous fungi [82]. 

## 5. Conclusions

In this work, it has been shown that (i) PO212 crude extracts (CE) have similar disease control activity as live PO212 conidia; (ii) PO212 expresses proteins and secondary metabolites in absence of pathogen and/or host; and (iii) PO212 growing under nitrogen stress conditions was able to excrete to the growth medium a range of extracellular proteins (mainly glycoside hydrolases, oxidases and peptidases) and secondary metabolites (*N*-(2-methyl-3-oxodec-8-enoyl) pyrrole, pyrogallol (1,2,3-trihydroxybenzene), and sorbicillin-related compound), which could partly explain the activity of PO212 CE against *Fusarium*. In the future, it would be interesting to work towards the synthetic production of the compounds and/or proteins excreted into the culture medium that are responsible for the activity of PO212 as inducer of resistance in plants, in addition to improving its efficiency.

## Figures and Tables

**Table 1:** Description of treatments applied in this work.

Treatment	Description	Application Dose	Assay
**PO212 ^1^**	PO212 conidia after growing into solid fermentation and dried in fluid-drier	1 × 107 conidia g^−1^ substrate	A1, A2, A3, A4
**aPO212 ^1,2^**	Autoclaved PO212 conidial suspension	1 × 10^7^ conidia g^−1^ substrate	A1
**SCC7 ^1^**	PO212 conidia after growing into Morton’s medium for 7 days	1 × 10^7^ conidia g^−1^ substrate	A1
**SCC14 ^1^**	PO212 conidia after growing into Morton’s medium for 14 days	1 × 10^7^ conidia g^−1^ substrate	A1
**CE7**	PO212 crude extract after growing into Morton’s medium for 7 days	60 mL kg^−1^ substrate	A1, A2, A3, A4
**CE14**	PO212 crude extracts after growing into Morton’s medium for 14 days	60 mL kg^−1^ substrate	A1
**aCE7 ^2^**	CE7 was autoclaved	60 mL kg^−1^ substrate	A1
**aCE14 ^2^**	CE14 was autoclaved	60 mL kg^−1^ substrate	A1
**CE7 1:1**	CE7 was mixed with SDW in a ratio of 1:1 (*v*/*v*)	60 mL kg^−1^ substrate	A2
**CE7 1:10**	CE7 was mixed with SDW in a ratio of 1:10 (*v*/*v*)	60 mL kg^−1^ substrate	A2
**CE7 1:50**	CE7 was mixed with SDW in a ratio of 1:50 (*v*/*v*)	60 mL kg^−1^ substrate	A2
**d-CE7**	CE7 was treated with proteinase K and DB at 50 °C for 1 h	60 mL kg^−1^ substrate	A3
**20f-CE7**	CE7 was kept at −20 °C for 24 h and then it was thawed under agitation to RT^3^	60 mL kg^−1^ substrate	A4
**80f-CE7**	CE7 was kept at −80 °C for 24h and then it was thawed under agitation to RT^3^	60 mL kg^−1^ substrate	A4
**DB**	Digestion buffer 5 × (250 mM Tris-HCl pH 7.5 and 25 mM CaCl_2_)	60 mL kg^−1^ substrate	A3
**Morton**	Morton’s B medium	60 mL kg^−1^ substrate	A1, A2, A3, A4
**C**	Control with SDW	60 mL kg^−1^ substrate	A1, A2, A3, A4

^1^ Conidia of *Penicillium rubens* strain 212 (PO212) were rehydrated with sterile distilled water (SDW) on a rotatory shaker at 150 rpm for 2 h. ^2^ Autoclaving was carried out during 30 min at 1 g cm^−2^ and 120 °C. ^3^ RT, room temperature.

**Table 2:** Activity of *Penicillium rubens* strain 212 (PO212) crude extracts (CE) and conidia (SCC) against disease caused by strain 1A of *Fusarium oxysporum* f.sp. *lycopersici* (FOL1A) in tomato plants at 28 days after transplanting and inoculation in growth chamber assays.

Treatment	Disease Severity (%)	Disease Reduction (%)	AUDPC
**PO212**	6.9 d	79.1	449.2 c
**aPO212**	33.9 ab	0	802.5 ab
**CE7**	12.7 d	63.6	432.8 c
**aCE7**	26.4 c	20.0	821.3 ab
**CE14**	17.1 d	48.2	551.3 c
**aCE14**	25.2 c	23.6	748.0 b
**SCC7**	31.8 b	3.6	805.5 ab
**SCC14**	37.5 a	0	1015.0 a
**Morton**	25.6 c	22.4	824.5 ab
**C1**	33.0 ab	-	782.5 ab
**MSE ^1^**	325.6		14852.8

Data are mean values of five replications (flasks) with four plants per flask. See Table 1 for description of treatments. aPO212, Autoclaved PO212 conidia, CE7, PO212 CE after growing into Morton’s medium for 7 days, aCE7, CE7 autoclaved, CE14, PO212 CE after growing into Morton’s medium for 14 days, aCE14, CE14 autoclaved, SCC7 PO212 conidia after growing into Morton’s medium for 7 days; SCC14 PO212 conidia after growing into Morton’s medium for 14 days, C1, plants treated with sterilized distilled water (SDW) and inoculated with FOL1A. Disease severity and the area under the disease progress curve (AUDPC) were estimated as described in Material and Methods. Means followed by the same letter in each column are not significantly different from each other (*p* ≥ 0.05) according to the Student–Newman–Keuls multiple range test. ^1^ MSE is mean squared error of analysis of variance.

**Table 3:** Effect of *Penicillium rubens* strain 212 (PO212) crude extracts (CE) and conidia (SCC) on the growth of tomato plants non- inoculated with strain 1A of *Fusarium oxysporum* f.sp. *lycopersici* (FOL1A) at 28 days after transplanting in growth chamber conditions assays.

Treatment	Nutritive Solution Consumption (mL Plant^−1^ Day^−1^)	Leaves Number Plant^−1^	Fresh Stem Weight (g Plant^−1^)	Root Weight (g Plant^−1^)
**PO212**	4.9	8.9 a	9.5 a	6.5 a
**aPO212**	4.9	7.8 b	8.2 b	5.5 cd
**CE7**	5.0	9.0 a	9.7 a	5.7 c
**aCE7**	4.8	9.1 a	8.0 b	5.4 cde
**CE14**	4.9	8.3 ab	8.1 b	5.7 c
**aCE14**	4.7	8.3 ab	8.1 b	5.4 cde
**SCC7**	5.0	8.3 ab	8.7 b	5.2 de
**SCC14**	4.7	8.3 ab	7.8 b	5.1 e
**Morton**	4.7	8.0 b	7.8 b	5.0 e
**C2**	3.0	7.9 b	7.8 b	6.2 b
**MSE ^1^**	3.6 NS	1.2	5.7	2.0

Data are mean values of five replications (flasks) with four plants per flask. See Table 1 for description of treatments. aPO212, Autoclaved PO212 conidia, CE7, PO212 CE after growing into Morton’s medium for 7 days, aCE7, CE7 autoclaved, CE14, PO212 CE after growing into Morton’s medium for 14 days, aCE14, CE14 autoclaved, SCC7 PO212 conidia after growing into Morton’s medium for 7 days; SCC14 PO212 conidia after growing into Morton’s medium for 14 days; C2, control plants treated with SDW and non-inoculated with FOL1A. Nutritive solution consumption, leaves number, fresh stem weight, and root weight were estimated as described in Material and Methods. Means followed by the same letter in each column are not significantly different from each other (*p* ≥ 0.05) according to the Student–Newman–Keuls multiple range test. ^1^ MSE is mean squared error of analysis of variance. NS, not significant.

**Table 4:** Evaluation of optimal concentration for CE7 (crude extract after growing into Morton’s medium for 7 days) application.

Treatment	Disease Severity (%)	Disease Reduction (%)	Disease Incidence (%)	AUDPC
**PO212**	33.1 c	32.2	40.0 b	636.8 c
**CE7**	33.4 c	31.6	62.5 a	629.3 c
**CE7 1:1**	41.7 b	14.5	64.1 a	698.1 bc
**CE7 1:10**	40.5 b	17.0	70.0 a	792.7 bc
**CE7 1:50**	51.6 a	0	68.8 a	1005.0 a
**Morton ^1^**	53.6 a	0	60.0 a	1027.5 a
**C1 ^2^**	48.8 a	-	65.0 a	808.9 b
**MSE ^3^**	143.4		2043.7	0.1

Data are mean values of two assays with ten replications (flasks) per treatments and four plants per replications. See Table 1 for description of treatments. *Penicillium rubens* strain 212 (PO212). Means followed by the same letter in each column are not significantly (*p* ≥ 0.05) different from each other according to the Student–Newman–Keuls multiple range test. ^1^ Morton, plants treated with Morton´s B medium; ^2^ C1, control plants treated with sterilized distilled water and inoculated with FOL1A. ^3^ MSE is mean squared error of analysis of variance.

**Table 5:** Effect of different concentrations of *Penicillium rubens* strain 212 (PO212) crude extract (CE7) on growth of strain 1A of *Fusarium oxysporum* f.sp. *lycopersici* (FOL1A).

Concentration (%)	Micelial Growth (mm day ^−1^)	Sporulation (Conidia Number Plate^−1^)	Germ Tubes Length (µm)	Germination (%)
**0 (Control)**	1.3	5.7 × 10^8^ (20.2) a	11.7 d	46.8 (27.9) c
**2.5**	ND	ND	15.9 c	74.5 (48.2) b
**12.5**	ND	ND	16.1 b	75.5 (49.1) ab
**25.0**	1.3	4.1 × 10^8^ (19.8) a	16.8 ab	77.8 (51.0) ab
**50.0**	1.3	1.5 × 10^8^ (18.8) b	17.6 a	78.5 (51.7) a
**80.0**	1.3	2.2 × 10^7^ (16.7) c	ND	ND
**MSE ^1^**	1.4 × 10^−4^ NS	2.9 × 10^17^ (11.9)	26.7	299.1

Sporulation data are mean value of two replicates with five plates per replicate. Germ tubes length data are mean of four replicates with twenty-five measures per repetition. Germination data are the mean of four replicates with three measures per replicate. Data in parentheses were subject to arcosen transformation to improve the homogeneity of variances before analysis. Means followed by the same letter in each column are not significantly (*p* ≥ 0.05) different from each other according to the Student–Newman–Keuls multiple range test. ^1^ MSE was the mean square error analysis. Control plates (0%) were treated with SDW. ND, non-determined. NS, not significant.

**Table 6:** Effect of CE7 fractions on the disease control caused by strain 1A of *Fusarium oxysporum* f.sp. *lycopersici* (FOL1A) and tomato plant growth.

Treatment	Disease Severity (%)	Disease Reduction (%)	AUDPC	Disease Incidence (%)	Leaf Number Plant^−1^	Stem Weight (g Plant^−1^)	Root Weight (g Plant^−1^)	Nutritive Solution Consumption (mL Plant^−1^ Day^−1^)
PO212 *	11.1 f	74.3	229.4 b	35.0 bc	6.3 b	1.0 e	1.2 c	1.7 d
**PO212**	0.0 g		0.0 c	0.0 c	8.2 a	3.3 a	1.9 a	5.1 b
CE7 *	16.3 ef	62.3	250.3 b	50.0 b	6.4 b	0.6 efg	0.8 e	1.5 d
**CE7**	0.0 g		0.0 c	0.0 c	7.0 b	2.7 b	1.7 b	3.9 bc
HMM-PF *	19.3 de	55.3	225.0 b	25.0 bc	6.1 b	0.7 efg	0.7 e	1.5 d
**HMM-PF**	0.0 g		0.0 c	0.0 c	6.2 b	2.4 c	1.3 c	4.3 bc
MMM-PF *	10.9 f	74.8	282.6 b	50.0 b	4.9 cd	0.7 ef	0.6 ef	1.2 d
**MMM-PF**	0.0 g		0.0 c	0.0 c	6.9 b	2.1 c	1.1 cd	6.8 a
Glu&Min *	25.4 cd	41.2	326.5 b	85.0 a	4.6 d	0.4 fgh	0.4 f	0.6 d
**Glu&Min**	0.0 g		0.0 c	0.0 c	6.7 b	1.5 d	0.8 e	4.3 bc
LMM-MF *	30.3 c	29.9	252.4 b	40.0 b	3.9 d	0.3 gh	0.4 f	0.7 d
**LMM-MF**	0.0 g		0.0 c	0.0 c	5.7 bc	1.5 d	0.9 de	3.0 c
Morton B *	49.7 a	0	471.5 a	83.3 a	6.2 b	0.5 fgh	0.6 ef	0.7 d
**Morton B**	0.0 g		0.0 c	0.0 c	6.1 b	1.7 d	1.1 c	3.6 c
C1 *	43.2 b		521.3 a	85.0 a	4.0 d	0.2 h	0.4 f	0.2 d
**C2**	0.0 g		0.0 c	0.0 c	6.9 b	2.0 c	1.1 cd	3.1 c
**MSE ^1^**	24.18		4092.89	355.03	0.45	0.06	0.02	0.61

Data are mean values of five replications (flasks) with four plants per replicate. Treatments with an asterisk (*) were plants inoculated with FOL1A, and treatments without an asterisk were plants non-inoculated with FOL1A. Treatments were: (i) PO212 (dried conidia of *Penicillium rubens* strain 212 from solid-state fermentation system, 10^7^ conidia g substrate^−1^), (ii) CE7 (PO212 crude extract after growth for seven days in Morton’s medium), (iii) HMM-PF, high molecular mass (MM) protein fraction >5 kDa, (iv) MMM-PF, middle MM protein fraction, 5–1 kDa, (v) Glu&Min, nutrients from culture medium (mainly glucose and minerals), (vi) LMM-MF, low MM metabolic fraction, (vii) Morton B, culture liquid medium, (viii) C1, plants treated with sterilized distilled water (SDW) and inoculated with FOL1A, (ix) C2, plants treated with SDW and noninoculated with FOL1A. Means followed by the same letter in each column are not significantly different from each other (*p* ≥ 0.05) according to the Student–Newman–Keuls multiple range test. ^1^ MSE is mean squared error of analysis of variance.

**Table 7:** Effect of freeze-drying of the high and middle molecular mass protein fractions (HMM-PF and MMM-PF) on the disease control caused by strain 1A of *Fusarium oxysporum* f.sp. *lycopersici* (FOL1A) and tomato plants growth.

Treatment	Disease Severity (%)	Disease Reduction (%)	AUDPC	Disease Incidence (%)	Leaf Number Plant^−1^	Stem Weight (g Plant^−1^)	Root Weight (g Plant^−1^)	Nutritive Solution Consumption (mL Plant^−1^ Day^−1^)
**PO212**	11.1 c	74.3	229.4 (5.4) b	35.0 bc	6.3 a	1.0 b	1.2 a	1.7 b
**CE7**	16.3 bc	62.3	250.3 (5.5) b	50.0 ab	6.4 a	0.6 c	0.8 c	1.5 b
**CE7L**	13.7 c	68.3	198.3 (5.3) b	50.0 ab	4.0 c	0.3 de	0.4 d	0.7 bc
**HMM-PF**	19.3 bc	55.3	225.0 (5.4) b	25.0 bc	6.1 a	0.7 c	0.7 c	1.5 b
**HMM-PF/L**	24.5 b	43.3	246.9 (5.5) b	45.0 ab	5.2 b	0.5 cd	0.4 d	1.0 bc
**MMM-PF**	10.9 c	74.8	282.6 (5.6) b	50.0 ab	4.9 bc	0.7 c	0.6 c	1.2 b
**MMM-PF/L**	11.3 c	73.8	286.8 (5.6) b	40.0 bc	4.7 bc	0.7 c	0.6 c	1.3 b
**Morton B**	49.7 a	0	471.5 (6.2) a	83.3 a	6.2 a	0.5 cd	0.6 c	0.7 bc
**C1**	43.2 a	0	521.3 (6.2) a	85.0 a	4.0 a	0.2 e	0.4 d	0.2 c
**C2**	0.0 d		0.0 (0.0) c	0.0 c	6.9 a	2.0 a	1.1 b	3.1 a
**MSE ^1^**	39.98		3642.67	499.30	0.26	0.02	0.01	0.30

Data are mean values of five replications (flasks) with four plants per flask. Treatments were: (i) PO212 (dried conidia of *Penicillium rubens* strain 212 from solid-state fermentation system, 10^7^ conidia g substrate^−1^), (ii) CE7, submerged crude extract of PO212 after growth for seven days in Morton’s medium, (iii) CE7L, crude extract of PO212 freeze-dried, (iv) HMM-PF (high molecular mass protein fraction) >5 kDa, (v) HMM-PF/L, HMM-PF freeze-dried, (vi) MMM-PF (middle molecular mass protein fraction) 5–1 kDa, (vii) MMM-PF,/L, MMM-PF freeze-dried, (viii) Morton B, culture liquid medium, (ix) C1, plants treated with SDW and inoculated with FOL1A, and (x) C2, plants treated with SDW and non-inoculated with FOL1A. Means followed by the same letter in each column are not significantly different from each other (*p* ≥ 0.05) according to the Student–Newman–Keuls multiple range test. ^1^ MSE is mean squared error of analysis of variance.

**Table 8:** Extracellular proteins identified (class, PO212 gene ID, and description) in the *Penicillium rubens* strain 212 (PO212) crude extract after growth for seven days in Morton’s medium (CE7).

Proteins	PO212 Gene ID	Function	MM ^1^ (kDa)	pI ^1^
**With Signal Peptide**				
Serine peptidase-type carboxypeptidase family 10	g9920	Proteolysis	57.6	5.4
Serine peptidase-type carboxypeptidase family 10	g9786	Proteolysis	61.4	4.9
Serine alkaline protease family 8	g5776	Endopeptidase activity	40.3	6.2
Flavin adenine dinucleotide binding	g7429	Oxidoreductase activity	61.6	8.5
Flavin adenine dinucleotide binding	g9078	Oxidoreductase activity	60.1	8.3
Flavin adenine dinucleotide binding	g6703	Oxidoreductase activity	48.4	4.6
GH family 17	g4058	Metabolic process	45.6	4.8
Ion calcio binding–1,2-α-mannosidase activity family 47	g3615	Metabolic process	56.9	5.1
Glucan endo-1,6-β-glucosidase family 5	g5409	Carbohydrate metabolic process	67.5	4.5
Hydrolase *O*-glycosyl compounds family 3	g9789	Carbohydrate metabolic process	52.0	5.2
Glucan 1,4-α-glucosidase family 15	g8238	Carbohydrate metabolic process	56.4	5.2
GH family 32	g3404	Carbohydrate metabolic process	77.5	4.9
1,3-β-glucanosil transferase family 72	g9967	Carbohydrate metabolic process	36.0	4.9
GH family 16	g9585	Carbohydrate metabolic process	35.8	6.7
GH family 43	g1771	Carbohydrate metabolic process	41.7	5.7
Uncharacterized protein	g4235	Uncharacterized protein	40.9	4.8
Uncharacterized protein	g9424	Uncharacterized protein	43.3	5.5
Others	g37	Transmembrane carrier	61.8	4.8
**Lacking Signal Peptide**				
GAPDH activity, NAD and NADP binding	g5740	Oxidoreductase activity	36.0	6.7
GH family 32	g4776	Carbohydrate metabolic process	62.5	5.3
ATP binding	g6856	Gamma-actin	59.5	8.9
ATP binding	g7817	Hydrolase and proton-transporting ATP synthase activity	61.8	5.7
ATP binding	g3421	Chaperonin	49.5	4.7
Others	g468	Putative cation/Cl^−^ transporter	130.9	6.6
Others	g7400	RNA binding	82.3	8.3
Others	g461	Transmembrane transporter activity	139.7	6.8
Others	g5657	Transcription initiation from RNA polymerase III promoter	69.2	5.4
Uncharacterized protein	g6156	Uncharacterized protein	30.7	10.4
Uncharacterized protein	g7827	Uncharacterized protein	40.3	6.6
Uncharacterized protein	g6729	Uncharacterized protein	129.1	6.7

^1^ Theoretical isoelectric point (pI) and MM were determined with Compute pI/MM tool (ExPASy) and the subcellular localization deduced with SignalP.

**Table 9:** Extracellular proteins identified (class, *Penicillium rubens* strain 212 (PO212) gene ID and description) in the high molecular mass protein fraction (HMM-PF).

Proteins	PO212 Gene ID	Function	MM ^1^ (kDa)	pI ^1^
**With Signal Peptide**				
Serine peptidase-type carboxypeptidase family 10	g9920	Proteolysis	61.8	4.8
Serine alkaline protease family 8	g5776	Endopeptidase activity	40.3	6.2
Flavin adenine dinucleotide binding	g9251	Oxidoreductase activity	50.3	5.5
Flavin adenine dinucleotide binding	g9078	Oxidoreductase activity	61.6	8.5
Flavin adenine dinucleotide binding	g6703	Oxidoreductase activity	60.1	8.3
GH family 17	g4058	Metabolism	48.4	4.6
Ion calcio binding–1,2-α-mannosidase activity family 47	g3615	Metabolism	56.4	5.2
Hydrolase *O*-glycosyl compounds family 3	g9789	Carbohydrate metabolic process	77.5	4.9
Glucan 1,4-α-glucosidase family 15	g8238	Carbohydrate metabolic process	52.0	5.2
GH family 16	g9585	Metabolism	36.0	4.9
GH family 43	g7971	Carbohydrate metabolic process	35.7	5.7
GH family 25	g6523	Carbohydrate metabolic process	23.8	5.8
Cell wall components	g7617	Structural component of the cell wall	16.2	5.3
	g3292	Structural component of the cell wall	14.6	4.6
**Lacking Signal Peptide**				
GAPDH activity, NAD and NADP binding	g5740	Oxidoreductase activity	36.0	6.7
ATP binding	g6856	Gamma-actin	41.7	5.7
ATP binding	g10134	ATP binding	69.5	5.1
ATP binding	g7817	Hydrolase and proton-transporting ATP synthase activity	59.5	8.9
Peptidase	g1273	Metallopeptidase activity	33.8	5.5
GH	g6997	Enoyl-[acyl-carrier-protein] reductase (NADH) activity	232.8	6.3
Others	g6816	Metal ion binding and ubiquitin-protein transferase activity	79.4	7.5
Others	g3420	Phospho relay sensor kinase activity	112.1	5.5
Uncharacterized protein	g10276	Uncharacterized protein	34.3	7.5

^1^ Theoretical isoelectric point (pI) and MM were determined with Compute pI/MM tool (ExPASy) and the subcellular localization deduced with SignalP.

## Supplementary Materials

The following supplementary materials are available online at https://www.mdpi.com/2309-608X/6/3/131/s1, Table S1: Proteins identified by mass spectrometry in the crude extract of *Penicillium rubens* strain 212 (PO212) after seven days of growth in Morton’s medium (CE7) through the proteome analysis platform Discoverer™ (version 1.4.1.14, Thermo Scientific). Table S2: Proteins identified by mass spectrometry in the high molecular mass (MM) protein fraction (HMM-PF) (>5 kDa) from crude extract of *Penicillium rubens* strain 212 (PO212) after seven days of growth in Morton’s medium (CE7) through the proteome analysis platform Discoverer™ (version 1.4.1.14, Thermo Scientific). Table S3: Proteins identified by mass spectrometry in the middle molecular mass (MM) protein fraction (MMM-PF) (5–1 kDa) from crude extract of *Penicillium rubens* strain 212 (PO212) after seven days of growth in Morton’s medium (CE7) through the proteome analysis platform Discoverer™ (version 1.4.1.14, Thermo Scientific). Figure S1: Schematic description of seedlings assay in growth chamber.
