# Supplementary material for: Exploring the Extracellular Macromolecular Composition of Crude Extracts of Penicillium rubens Strain 212 for Elucidation Its Mode of Action as a Biocontrol Agent
Source: J Fungi (Basel). 2020 Aug 10;6(3):131. doi: 10.3390/jof6030131 (PMC7559091; doi:10.3390/jof6030131)
Supplement: Supplementary file 1 [file jof-06-00131-s001.pdf]

Journal of *Fungi*

MDPI

Supplementary Materials: Exploring the Extracellular Macromolecular Composition of Crude Extracts of *Penicillium rubens* Strain 212 for Elucidation Its Mode of Action as a Biocontrol Agent

Table S1. Proteins identified by mass spectrometry in the crude extract of *Penicillium rubens* strain 212 (PO212) after seven-days of growth in Morton's medium (CE7) through the Proteome analysis platform Discoverer™ (version 1.4.1.14, Thermo Scientific).

Access NCBI[1]	Gene PRwis[2]	Score[3]	Coverage[4]	Proteins /Peptides	aa	MM (kDa)	pI	UniProtKB	Gene PO212[5]	SignalP[6]
XP_002559154.1	Pc13g07230	2.8	0.8	1 / 1	1192	131.0	6.6	B6H3S2	g468	no
XP_002564558.1	Pc22g05230	2.7	3.4	1 / 1	267	30.7	10.4	B6HUD6	g6156	no
XP_002565602.1	Pc22g16890	2.6	1.5	1 / 1	724	82.3	8.3	B6HRZ9	g7400	no
XP_002559146.1	Pc13g07150	2.1	0.8	1 / 1	1275	139.7	6.8	B6H3Q2	g461	no
XP_002568563.1	Pc21g15530	3.6	2.4	1 / 1	620	69.2	5.4	B6HJV1	g5657	no
XP_002557212.1	Pc12g03270	2.9	2.3	1 / 1	351	40.3	6.6	B6H0V6	g7827	no
XP_002563795.1	Pc20g13140	2.0	0.9	1 / 1	1167	129.1	6.7	B6HGQ1	g6729	no
XP_002568470.1	Pc21g14560	4.5	4.2	1 / 1	337	36.0	6.7	B6HI59	g5740	no
XP_002560026.1	Pc14g00310	3.9	2.9	1 / 1	580	62.5	5.3	B6H5K1	g4776	no
XP_002563649.1	Pc20g11630	3.2	2.4	1 / 3	554	59.5	8.9	B6HG97	g6856	no
XP_002557222.1	Pc12g03370	3.1	1.5	1 / 1	588	61.8	5.7	B6H0W6	g7817	no
XP_002561413.1	Pc16g11070	4.3	3.5	1 / 1	454	49.5	4.7	B6H9L7	g3421	no
XP_002564950.1	Pc22g09380	2.9	3.5	1 / 1	343	36.0	4.9	B6HU11	g9967	Yes
XP_002569288.1	Pc21g23210	4.1	4.3	1 / 1	325	35.8	6.7	B6HI97	g9585	Yes
XP_002557900.1	Pc12g10800	10.4	9.9	1 / 1	375	41.7	5.7	B6H102	g1771	Yes
XP_002561014.1	Pc16g06800	3.5	2.8	1 / 1	392	40.9	4.8	B6H7P7	g4235	Yes
XP_002565696.1	Pc22g17870	14.6	12.5	1 / 1	401	43.3	5.5	B6HTP0	g9424	Yes
XP_002566382.1	Pc22g24940	25.0	12.7	1 / 3	559	61.8	4.8	B6HRN4	g37	Yes
XP_002565008.1	Pc22g09990	3.0	2.1	1 / 4	524	57.6	5.4	B6HV80	g9920	Yes
XP_002563517.1	Pc20g10240	10.7	6.6	1 / 1	561	61.4	4.9	B6HFM4	g9786	Yes
XP_002568432.1	Pc21g14160	5.9	6.0	1 / 2	398	40.3	6.2	B6HHN9	g5776	Yes
XP_002565565.1	Pc22g16510	3.6	2.3	1 / 3	564	61.6	8.5	B6HRI4	g7429	Yes
XP_002567314.1	Pc21g02500	3.1	2.5	1 / 1	559	60.1	8.3	B6HK00	g9078	Yes
XP_002563824.1	Pc20g13440	4.1	2.6	1 / 1	494	48.4	4.6	B6HGY3	g6703	Yes
XP_002560249.1	Pc15g00210	3.7	2.2	1 / 1	417	45.6	4.8	B6H6G8	g4058	Yes

XP_002562102.1	Pc18g02600	2.3	2.7	1 / 1	622	67.5	4.5	B6HCY2	g5409	Yes
XP_002561396.1	Pc16g10900	7.5	5.0	1 / 1	736	77.5	4.9	B6H9G4	g3404	Yes
XP_002563511.1	Pc20g10170	8.3	6.4	1 / 2	488	52.0	5.2	B6HFL8	g9789	Yes
XP_002564229.1	Pc22g01850	8.1	6.1	1 / 2	510	56.4	5.2	B6HPB5	g8238	Yes
XP_002561715.1	Pc16g14170	3.0	3.0	1 / 2	538	56.9	5.1	B6H9W4	g3615	Yes

[1]Access NCBI, access number of identified genes in *Penicillium rubens* Wisconsin strain 54-1255; [2]Gene PRwis, identified gene in *Penicillium rubens* Wisconsin strain 54-1255; [3]Score, statistical parameter for identification reliability; [4]Coverage, percentage of identified protein sequence; [5]Homologe genes identified in PO212 genome; [6]SignalP, Signal Peptide was predicted based on SignalP 5.0. Amino acids number (aa), molecular mass (MM) and isoelectric point (pI) of identified proteins were determined theoretically (https://www.expasy.org).

Table S2. Proteins identified by mass spectrometry in the high molecular mass (MM) protein fraction (HMM-PF) (>5 kDa) from crude extract of *Penicillium rubens* strain 212 (PO212) after seven-days of growth in Morton's medium (CE7) through the Proteome analysis platform Discoverer™ (version 1.4.1.14, Thermo Scientific).

Access NCBI[1]	Gene PRwis[2]	Score[3]	Coverage[4]	Proteins /Peptides	aa	MM (kDa)	pI	UniProtKB	Gene PO212[5]	SignalP[6]
XP_002563692.1	Pc20g12060	3.3	2.3	1 / 1	703	79.4	7.5	B6HDL8	g6816	no
XP_002561412.1	Pc16g11060	2.7	1.1	1 / 1	1025	112.1	5.5	B6H9L6	g3420	no
XP_002565183.1	Pc22g12390	7.6	17.1	3 / 1	305	34.3	7.5	B6HRI1	g10276	no
XP_002568470.1	Pc21g14560	4.8	4.2	1 / 1	337	36.0	6.7	B6HI59	g5740	no
XP_002563649.1	Pc20g11630	11.6	9.9	1 / 3	375	41.7	5.7	B6HG97	g6856	no
XP_002565071.1	Pc22g11240	4.3	2.5	1 / 1	635	69.5	5.1	B6HG97	g10134	no
XP_002557222.1	Pc12g03370	3.6	2.4	1 / 1	554	59.5	8.9	B6H0W6	g7817	no
XP_002557356.1	Pc12g05090	3.8	4.5	1 / 1	311	33.8	5.5	B6GYU3	g1273	no
XP_002568119.1	Pc21g10870	2.4	0.4	1 / 1	2119	232.8	6.3	B6HJF0	g6997	no
XP_002568432.1	Pc21g14160	6.0	4.8	1 / 1	398	40.3	6.2	B6HHN9	g5776	Yes
XP_002565008.1	Pc22g09990	29.6	12.7	1 / 4	559	61.8	4.8	B6HV80	g9920	Yes
XP_002563824.1	Pc20g13440	28.1	12.5	1 / 4	559	60.1	8.3	B6HGY3	g6703	Yes
XP_002567314.1	Pc21g02500	11.9	6.7	1 / 3	564	61.6	8.5	B6HK00	g9078	Yes
XP_002567557.1	Pc21g05110	4.0	2.3	1 / 1	471	50.3	5.5	B6HNK6	g9251	Yes
XP_002569288.1	Pc21g23210	8.9	7.6	1 / 2	343	36.0	4.9	B6HI97	g9585	Yes
XP_002557032.1	Pc12g01330	5.1	5.4	1 / 1	331	35.7	5.7	B6GYS2	g7971	Yes
XP_002564022.1	Pc20g15500	4.5	5.3	1 / 1	225	23.8	5.8	B6HE04	g6523	Yes
XP_002564229.1	Pc22g01850	41.4	21.5	1 / 8	488	52.0	5.2	B6HPB5	g8238	Yes
XP_002563511.1	Pc20g10170	23.7	9.9	1 / 4	736	77.5	4.9	B6HFL8	g9789	Yes
XP_002560249.1	Pc15g00210	12.1	5.3	1 / 2	494	48.4	4.6	B6H6G8	g4058	Yes
XP_002561715.1	Pc16g14170	22.4	13.5	1 / 5	510	56.4	5.2	B6H9W4	g3615	Yes
XP_002565353.1	Pc22g14290	4.9	7.4	1 / 1	163	16.2	5.3	B6HUJ4	g7617	Yes
XP_002568829.1	Pc21g18350	4.2	10.3	1 / 1	146	14.6	4.6	B6HNW2	g3292	Yes

[1]Access NCBI, access number of identified genes in *Penicillium rubens* Wisconsin strain 54-1255; [2]Gene PRwis, identified gene in *Penicillium rubens* Wisconsin strain 54-1255; [3]Score, statistical parameter for identification reliability; [4]Coverage, percentage of identified protein sequence; [5]Homologe genes identified in PO212 genome; [6]SignalP, Signal Peptide was predicted based on SignalP 5.0. Amino acids number (aa), MM and isoelectric point (pI) of identified proteins were determined theoretically (https://www.expasy.org).

Table S3. Proteins identified by mass spectrometry in the middle molecular mass (MM) protein fraction (MMM-PF) (5-1 kDa) from crude extract of *Penicillium rubens* strain 212 (PO212) after seven-days of growth in Morton's medium (CE7) through the Proteome analysis platform Discoverer™ (version 1.4.1.14, Thermo Scientific).

Access NCBI[1]	Gene PRwis[2]	Score[3]	Coverage[4]	Proteins /Peptides	aa	MM (kDa)	pI	UniProtKB	Gene PO212[5]	SignalP[6]
XP_002563649.1	Pc20g11630	11.6	9.9	1/3	375	41.7	5.7	B6HG97	g6856	no
XP_002568042.1	Pc21g10070	4.8	2.7	1/1	518	55.2	5.4	B6HI25	g7065	no

[1]Access NCBI, access number of identified genes in *Penicillium rubens* Wisconsin strain 54-1255; [2]Gene PRwis, identified gene in *Penicillium rubens* Wisconsin strain 54-1255; [3]Score, statistical parameter for identification reliability; [4]Coverage, percentage of identified protein sequence; [5]Hologe genes identified in PO212 genome; [6]SignalP, Signal Peptide was predicted based on SignalP 5.0. Amino acids number (aa), MM and isoelectric point (pI) of identified proteins were determined theoretically (https://www.expasy.org).

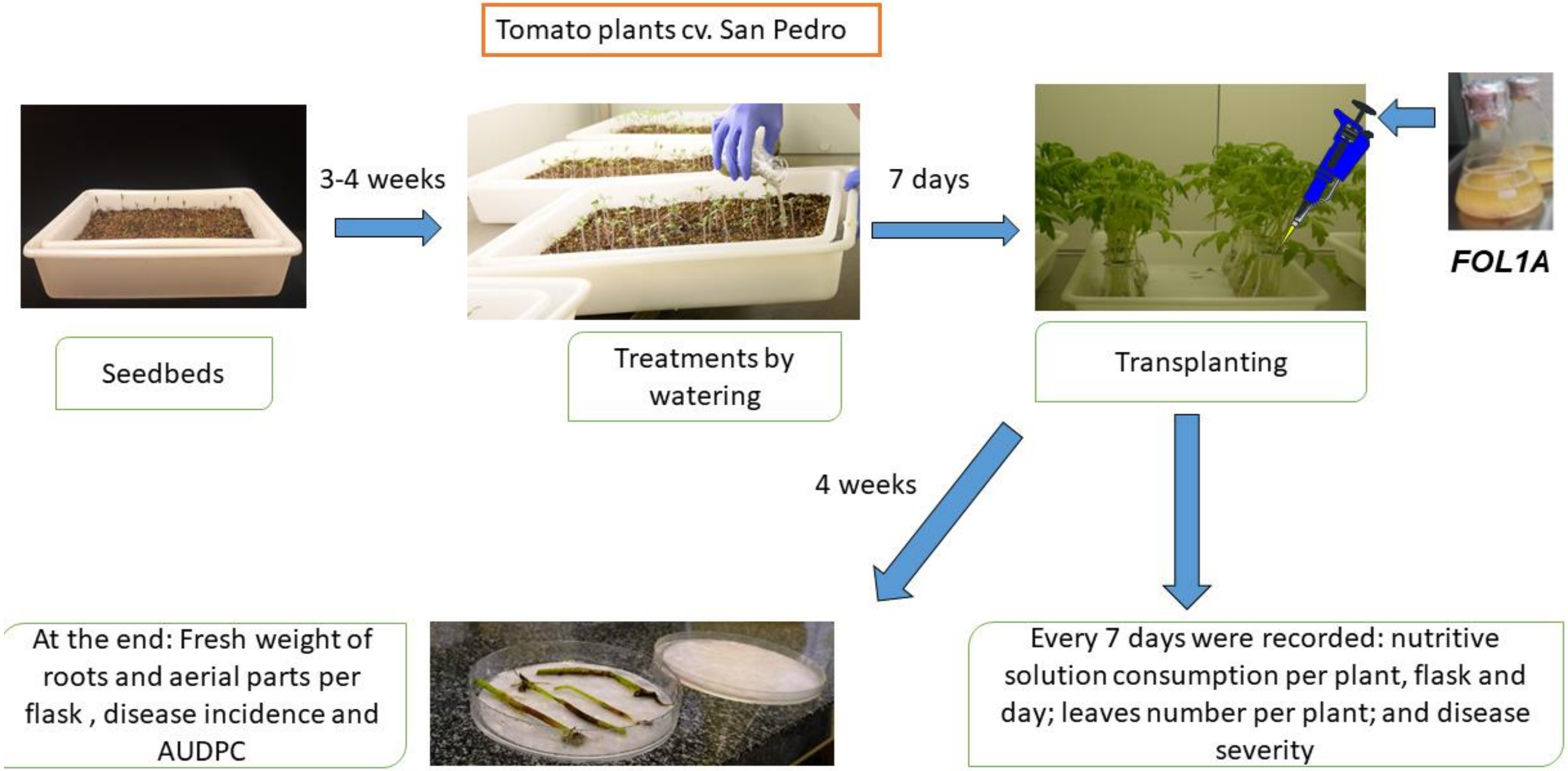

Figure S1. Schematic description of seedlings assay in growth chamber. Assays in growth chambers (22–28 °C, under fluorescent light (100 μE m−2 s−1 and 16 h photoperiod) and 80–100% relative humidity.
